# Deep learning for atrial electrogram estimation: toward non-invasive arrhythmia mapping using variational autoencoders

**DOI:** 10.3389/fphys.2025.1720244

**Published:** 2026-01-12

**Authors:** Miriam Gutiérrez-Fernández, K. López-Linares, C. Fambuena-Santos, Maria S. Guillem, Andreu M. Climent, Ó. Barquero-Pérez

**Affiliations:** 1 Signal Theory and Communications Dpt., EIF, Universidad Rey Juan Carlos, Fuenlabrada, Spain; 2 Vicomtech Foundation, Basque Research and Technology Alliance (BRTA), San Sebastián, Spain; 3 eHealth Group, Bioengineering Area, Biogipuzkoa Health Research Institute, San Sebastián, Spain; 4 ITACA Institute, Universitat Politècnica de València, València, Spain

**Keywords:** atrial fibrillation, body surface potential mapping, deep learning, inverse problem, variational autoencoder

## Abstract

**Background:**

Non-invasive estimation of intracardiac electrograms (EGMs) from body surface potential measurements (BSPMs) could reduce reliance on invasive mapping and enable safer, patient-specific characterization of atrial arrhythmias. Conventional inverse problem formulations, such as Tikhonov regularization, are limited by ill-posedness, sensitivity to anatomical inaccuracies, and low spatial resolution.

**Objective:**

In this work, we propose a dual-branch deep learning (DL) architecture based on a variational autoencoder (VAE) to directly reconstruct atrial EGMs from BSPMs.

**Methods:**

A dataset of 680 BSPM-EGM pairs was generated using biatrial computational models simulating a wide spectrum of rhythms, including sinus rhythm, atrial fibrillation (AF), ectopic activity, and fibrotic substrates. The network learns a shared latent representation of BSPMs, simultaneously optimized for BSPM self-reconstruction and EGM prediction. Performance was assessed across two phases: a baseline dataset with well-represented rhythms (sinus and multirotor AF), and an extended dataset with rarer arrhythmic classes. Evaluation employed multiple temporal and spectral metrics, as well as spatial voltage and phase mapping.

**Results:**

Results show that stratified training yielded the most balanced performance, particularly in AF, with improved correlation, peak detection precision, and spectral coherence compared to baseline and regularized models. Against the zero-order Tikhonov method, the proposed DL model preserved waveform morphology and spectral content more faithfully across rhythm types.

**Conclusions:**

These findings demonstrate that non-invasive, data-driven EGM reconstruction is feasible and can capture physiologically relevant temporal and spatial dynamics. By providing more coherent functional information from BSPMs, DL-based approaches may support individualized diagnosis and guide ablation strategies in atrial arrhythmia care.

## 1 Introduction

Atrial fibrillation (AF) is the most common sustained arrhythmia worldwide, affecting over 33 million people and contributing to significant morbidity and mortality, especially due to stroke and heart failure. It represents a major and growing global health burden. Persistent AF, a subtype where episodes last longer than 7 days or require intervention to stop, is more resistant to treatment and associated with greater atrial remodeling, symptom severity, and risk of complications. The rising prevalence of AF, driven by aging and comorbidities, highlights the need for improved detection and management strategies (Van Gelder et al., 2024; Gómez-Doblasa et al., 2016; Elliott et al., 2023).

Three-dimensional (3D) anatomical mapping systems have become the foundation for guiding electrophysiological procedures in arrhythmia, particularly in AF treatment. These systems allow real-time visualization of cardiac structures and integrate intracardiac electrical signals to generate activation, voltage, and conduction maps. Such maps help clinicians identify regions that may sustain or trigger arrhythmias, commonly referred to as arrhythmogenic substrates (Knackstedt et al., 2008).

In catheter ablation, targets can be broadly classified into two categories: anatomical targets, which are predefined by cardiac structures and represented by mapping systems, and substrate-based (functional) targets, which are identified through the electrophysiological information obtained from these systems. Anatomical targets are predefined structural landmarks such as pulmonary vein ostia in AF, where ablation is applied based on known anatomical associations with arrhythmic circuits. These locations are often selected regardless of the specific patient’s electrophysiological conditions (Tsyganov et al., 2015). In contrast, functional or substrate-based ablation seeks to identify patient-specific regions with abnormal electrophysiological characteristics. Electrophysiologists rely on advanced electroanatomical mapping tools and a range of functional indicators, including areas of slowed conduction velocity (Zheng et al., 2017), low-voltage regions indicative of scar or fibrosis (Bailin et al., 2011), and zones of high dominant frequency associated with rapid reentrant activity (Atienza et al., 2009). These indicators reflect underlying conduction abnormalities and guide a more tailored approach to ablation, particularly in complex arrhythmias like persistent AF, where the arrhythmic substrate is not limited to fixed anatomical locations (Pappone and Santinelli, 2006).

There remains no clear consensus on whether catheter ablation should be primarily guided by anatomical or functional criteria, particularly in complex cases. In clinical practice, particularly in persistent AF, empirical anatomical-based lesion sets, including pulmonary vein isolation, posterior left atrial wall isolation, and vein of Marshall interventions, are still frequently performed, despite their added complexity, potential risks (La et al., 2021) and limited long-term success rates in treating complex arrhythmia (Goldberger, 2017). This is largely due to the limited spatial resolution and reliability of current functional mapping technologies, which difficults the electrophysiologist’s ability to accurately determine arrhythmogenic tissue. As a result, many electrophysiologists remain cautious about relying exclusively on functional indicators and continue to favor anatomical approaches (Knackstedt et al., 2008).

Furthermore, although invasive electroanatomical mapping remains the classic benchmark for localizing arrhythmic substrates, it suffers from several critical limitations that restrict its scalability and precision when representing functional targets. First, the accuracy of invasive mapping is inherently constrained by spatial sampling density; catheters can only record from a limited number of sites at a time, which can lead to under-sampling of critical regions (Knackstedt et al., 2008). Second, high-resolution maps require extensive catheter manipulation and prolonged procedural time, increasing patient risk and operator dependency. Third, even with advanced mapping systems, it remains challenging to reconstruct global activation patterns or capture transient arrhythmic behavior, especially in AF where the electrical activity is highly dynamic (Cuculich et al., 2010), (R et al., 2020). Lastly, invasive mapping provides limited insight into the epicardial surface, which may harbor critical components of arrhythmia circuits inaccessible from the endocardium. These limitations have catalyzed the search for non-invasive solutions, most notably, electrocardiographic imaging (ECGI).

ECGI is a non-invasive technique that reconstructs electrical activity happening on the heart’s surface (epicardium) by combining body surface potential measurements (BSPMs) recordings with patient-specific anatomical data, typically obtained from computed tomography or magnetic resonance scans. By solving the electrocardiography inverse problem, commonly leveraging regularization techniques like Tikhonov, it enables the estimation of epicardial potentials, activation times, and other electrophysiological parameters, offering a promising route toward safer, faster, and more comprehensive arrhythmia characterization. ECGI’s principal advantage over invasive mapping lies in its capacity to deliver a global, beat-by-beat perspective of cardiac electrical activity, offering highly valuable complementary information for the characterization of AF dynamics (Knackstedt et al., 2008).

Several landmark ECGI studies have demonstrated the potential of non-invasive mapping for atrial arrhythmias, including the early work of Rudy and colleagues on reconstructing epicardial potentials (Rudy and Burnes, 1999), and the clinical studies by Haissaguerre, Rodrigo, and Salinet showing the feasibility of identifying activation patterns and AF drivers from the body surface (Haissaguerre et al., 2014; R et al., 2020; Salinet et al., 2021). Building upon this framework, recent studies have enhanced ECGI physiological interpretability by incorporating advanced computational techniques. Figuera et al. (Figuera et al., 2016) demonstrated how various regularization approaches, including spatial and temporal constraints, can improve the stability and detail of ECGI reconstructions during AF, reinforcing their potential for clinical use. Furthermore, Cámara-Vázquez et al. (Cámara-Vázquez et al., 2021a) proposed a multimodal strategy that integrates intracardiac signals with surface potentials, showing that this hybrid approach provides more detailed and reliable global maps of AF.

However, this technique still faces significant challenges, including the need for detailed anatomical meshes, high sensitivity to geometric inaccuracies, and reliance on complex inverse problem formulations that are inherently ill-posed and computationally demanding (Hernández-Romero et al., 2023). As a result, ECGI reconstructions are highly susceptible to noise and can often lead to overly smoothed EGM maps that lack sufficient detail to accurately identify AF-related functional arrhythmogenic regions, limiting their utility in guiding ablation procedures.

This exacerbated in ECGI targeting atria signals: unlike ventricular ECGI, which benefits from larger signal amplitudes, more synchronous activation, and decades of methodological refinement, atrial ECGI must deal with intrinsically weaker signals, highly variable propagation patterns, and complex anatomical substrates. Atrial activity, particularly during AF, exhibits multiple simultaneous wavefronts, rapid cycle length variability, and strong anisotropy, all of which exacerbate the ill-posedness of the inverse problem (Cheniti et al., 2019). As demonstrated in prior works, atrial ECGI solutions based on traditional approaches like ZOT are highly sensitive to small geometric inaccuracies, noise, and the choice of regularization, often resulting in over-smoothed electrograms with limited value for functional mapping (Salinet et al., 2021).

Recent advances in artificial intelligence (AI), and particularly in deep learning (DL), offer a promising opportunity to rethink ECGI from a data-driven perspective. While traditional ECGI approaches rely on algebraic formulations and regularization-based techniques to solve the inverse problem, recent years have seen a growing number of studies exploring DL for non-invasive EGM estimation as an alternative framework. These data-driven methods aim to learn direct mappings from BSPMs to epicardial activity, potentially overcoming the limitations of conventional techniques related to ill-posedness, sensitivity to anatomical inaccuracies, and computational complexity.

Various DL architectures have been proposed to address non-invasive EGM estimation. Convolutional Neural Networks (CNNs) have shown strong performance in capturing spatial patterns in torso potential maps (Cámara-Vázquez et al., 2021b; Chen et al., 2022; Lack et al., 2021). Recurrent architectures have demonstrated effectiveness in reconstructing local electrograms (EGMs) with preserved spectral content, particularly in the context of AF (Gutiérrez-Fernández et al., 2023). In (Chen et al., 2022), they used a model based on CNN and LSTM trained on ventricle pig data to obtain EGM estimates, obtaining correlation coefficient of 0.74, although they did not tested their model in atria or AF. In (Wang et al., 2025) they used a Pix2Pix network and a cosine similarity specific loss function to reconstruct 2D heart surface potential maps during ventricle pacing, obtaining a mean of 0.64 correlation coefficient.

Other approaches incorporate anatomical priors or physics-inspired constraints to enhance biological plausibility and improve generalization in data-limited settings. In (X et al., 2022), they integrate the physics law of the cardiac electrical wave propagation within DL architecture to predict heart surface potentials (Tenderini et al., 2022). proposes neural networks that incorporate partial differential equations to reconstruct epicardial potentials using a small amount of clinical data.

Overall, the literature remains predominantly focused on ventricular data, likely due to its more favorable conditioning and higher signal quality. In contrast, DL approaches targeting atrial signals are comparatively scarce, despite the substantial and growing potential of ECGI for characterizing AF, whose mechanisms are still not fully understood, and the fact that current rhythm-management outcomes remain far from optimal (Goldberger, 2017).

Super-resolution techniques have been extensively studied in the field of computer vision in biomedical applications, particularly for reconstructing high-resolution medical images from low-quality or downsampled inputs (Heydari and Mehmood, 2020; Chira et al., 2022; Xiao et al., 2025). Among the most effective models for this task are convolutional variational autoencoders (VAEs), which learn nonlinear mappings between degraded inputs and their high-resolution counterparts by embedding them into a low-dimensional latent space. This architecture has proven useful not only for image enhancement but also as a framework for solving a wide range of inverse problems, where reconstructing missing or unobservable data from indirect measurements is essential (Go et al., 2019; Prost et al., 2023). Inspired by these developments, VAEs have recently been adapted for noninvasive EGM estimation, offering a data-driven alternative for spatial and temporal signal estimation in complex biological systems. In particular, Bacoyannis et al. integrate BSPMs and anatomical priors to perform multimodal estimation of epicardial activation sequences using a conditional VAE, demonstrating their potential to model cardiac activation patterns beyond traditional inverse problem formulations in the ventricles, although their model was not tested in atria and still requires anatomical prior information (Bacoyannis et al., 2022).

Despite the fact that AF ablation ultimately aims to identify and treat arrhythmogenic substrates, achieving this goal depends on reliable functional information extracted from intracardiac EGMs. However, current invasive electroanatomical mapping provides incomplete and often inconsistent ground truth, particularly in AF, making direct substrate identification from BSPMs currently infeasible for data-driven methods. Consequently, reconstructing high-fidelity intracavitary EGMs represents a necessary intermediate step: only from accurate EGMs can functional markers such as dominant frequency, voltage, activation time, or phase singularities be computed with clinical reliability. Motivated by this, the present study focuses specifically on the non-invasive reconstruction of EGMs as a foundational component of future substrate-based AF mapping.

We propose a deep learning model capable of estimating EGMs from BSPMs in the atria without requiring prior anatomical knowledge or geometrical information. Specifically, the approach consists of a dual-branch deep learning model based on a shared latent representation. First, an encoder extracts spatiotemporal features from BSPMs and compresses them into a latent space. This latent space is simultaneously used by two branches: (1) a decoder branch that reconstructs BSPMs, serving as an auxiliary self-reconstruction task to regularize and enrich the latent representation, and (2) an EGM estimation branch, where the latent space is processed by an LSTM-based module to generate intracardiac EGMs. We evaluated the model under different training regimes, including the application of regularization and data stratification techniques. Performance was assessed across a range of cardiac rhythms, including both simple rhythms and more complex arrhythmias such as AF. Additionally, the model’s generalizability was tested on a limited subset of additional complex cases, including fibrosis and ectopic beats.

The manuscript is structured as follows: the generation of the training dataset, dataset description, methods employed, and evaluation metrics are detailed in the Materials and Methods section. In the Results section, both quantitative and qualitative findings are presented and discussed. Finally, the Conclusions section summarizes key take-home messages and outlines directions for future work.

## 2 Dataset

This section details the dataset construction, from EGM to BSPMs, according to different patient groups. Since training a DL model requires large volumes of annotated data, and direct intracardiac measurements are scarce in clinical practice, we relied on synthetic simulations to generate realistic ground-truth EGMs. These EGMs were produced using computational atrial models that replicate a wide range of physiological and pathological activation patterns, including sinus rhythm, ectopic activity, and complex arrhythmias such as AF and flutter. Then, to compute the corresponding BSPMs, the forward problem of electrocardiography was solved using realistic torso geometries and the simulated EGMs.

### 2.1 EGM signal simulation - Atrial tissue modelling

A total of 68 EGMs, considered as unique patients, were generated using a realistic computational model of the atria comprising 2048 nodes that define the atrial geometry. The atrial tissue simulation model was originally developed by (Pedrón-Torrecilla et al., 2016) and was kindly shared with the authors for this study. The atrial model represents the tissue as a simplified bilayer structure - composed of the endocardium and epicardium - following the methodology described in (Rodrigo et al., 2017). The dataset includes a wide range of electrical propagation patterns, including sinus rhythm, functional reentries, stable rotors or drivers, ectopic wavefront propagation, and varying degrees of fibrosis and conduction heterogeneity.

The action potential of each node was simulated using a modified version of the Courtemanche human atrial cell model, incorporating an $I_{K,ACh}$ formulation. The transmembrane potential dynamics are governed by Equation 1:

$$\frac{\partial V_{m}}{\partial t}=-\frac{I_{\mathrm{ion}}}{C_{m}}$$ (1)

where $\frac{\partial V_{m}}{\partial t}$ is the transmembrane potential at each instant, $I_{\mathrm{ion}}$ the transmembrane ionic current and $C_{m}$ the cell membrane capacitance. To simulate tissue-level propagation, the model incorporates intercellular coupling through gap junctions as described in Equation 2:

$$\frac{\partial V_{k}}{\partial t}=-\frac{I_{\mathrm{ion}}}{C_{m}}-\overset{N}{\underset{i=1}{\sum }}D_{K,i}\frac{V_{k}-V_{i}}{d_{k,i}^{2}}$$ (2)

Here $V_{k}$ and $V_{i}$ denote the transmembrane potentials at nodes k and i , respectively; $D_{k,i}$ is the diffusion coefficient between those nodes; and $d_{k,i}$ is their Euclidean distance. Given the anisotropic conduction properties of atrial tissue, where conduction velocity is higher along fiber orientation (longitudinal) and lower across it (transversal), the diffusion coefficient is calculated in Equation 3:

$$D_{K,i}=D_{\mathrm{long}}\cdot cos^{2}\alpha +D_{\mathrm{trans}}\cdot sin^{2}\alpha$$ (3)

with α being the angle between the fiber direction and the axis connecting nodes k and i , $D_{\mathrm{long}}$ and $D_{\mathrm{trans}}$ the longitudinal and transverse diffusion coefficients.

Simulated EGMs were obtained from the transmembrane potentials by applying the standard quasi-static forward formulation given in Equation 4:

$$EGM=\underset{\vec{r}}{\sum }\left(\frac{\vec{r}}{r^{3}}\right)\cdot \nabla V_{m},$$ (4)

with the potentials evaluated at measurement points located 1 mm above the epicardial surface. The computation was based on a homogeneous, unbounded, quasi-static conductive medium model.

Because the transmembrane potentials are defined over a sparse 3D mesh, the spatial gradient $\nabla V_{m}$ was estimated using a second-order polynomial interpolation, based on the method from (Lawson, 1984), defined in Equation 5:

$$\begin{array}{cc} \nabla V_{m}=V_{m,i}-V_{m,j} & =c_{1}x+c_{2}y+c_{3}z+c_{4}x^{2}+c_{5}y^{2}\\ & \;+c_{6}z^{2}+c_{7}zy+c_{8}yz+c_{9}xz \end{array}$$ (5)

where $V_{m,i}$ and $V_{m,j}$ are the transmembrane potentials at neighboring nodes i and j , x , y , and z are the Cartesian coordinate differences between them, and $c_{1}$ to $c_{9}$ are the interpolation coefficients determined using a least-squares fit to at least nine neighboring nodes.

For fibrotic areas, a percentage of nodes were randomly deactivated according to the desired propagation pattern.

AF was induced using a standard S1–S2 pacing protocol, in which a train of regular conditioning stimuli (S1) is followed by a premature extra stimulus (S2). The early S2 stimulus creates conduction heterogeneities that promote wavebreak and rotor formation, leading to sustained AF (Pedrón-Torrecilla et al., 2016).

A detailed table with modelling parameters and geometries has been provided in Supplementary Table S5 from Supplementary Material.

### 2.2 Forward problem: body surface potential computation

The epicardial potentials $U_{A}$ obtained from the atrial model were used to compute the corresponding body-surface potentials $U_{T}$ by solving the forward problem of electrocardiography. This was achieved using the Boundary Element Method (BEM), which yields the linear relationship in Equation 6:

$$U_{T}=M\;U_{A}$$ (6)

where M is the transfer matrix mapping potentials on the atrial surface A to the torso surface T . Both surfaces were represented as triangular meshes, with each triangle defined by the coordinates of its three vertices. The transfer matrix M was computed using the classical single- and double-layer BEM formulation, expressed in Equation 7:

$$M={\left[D_{TT}-G_{TA}\;G_{AA}^{-1}\;D_{AT}\right]}^{-1}\cdot \left[G_{TA}\;G_{AA}^{-1}\;D_{AA}-D_{TA}\right]$$ (7)

In this formulation, $D_{XY}$ denotes the coefficient matrix describing the contribution of the potential on surface Y to the potential on surface X , while $G_{XY}$ denotes the matrix describing the contribution of the voltage gradient on surface Y to the potential on surface X . If surface Y contains $N_{Y}$ nodes and surface X contains $N_{X}$ nodes, then $D_{XY}$ and $G_{XY}$ are matrices of dimension $N_{X}\times N_{Y}$ .

For each EGM, BSPMs were calculated over 10 different torso geometries and transfer matrices, each comprising between 659 and 3,970 nodes. To emulate a realistic 64-electrode clinical setup in Supplementary Figures S1, 2 (Pedrón-Torrecilla et al., 2016), we identified, for each torso model, the surface nodes closest to the anatomical locations of the clinical electrode landmarks. These nodes were then used as surrogate electrode positions, effectively simulating a 64-electrode vest. This procedure follows the methodology established in our previous work (Cámara-Vázquez et al., 2021a; Gutiérrez-Fernández et al., 2023; Gutiérrez-F et al., 2024).

Transfer matrices for generating realistic torso models were constructed from MRI-derived torso and atrial geometries, assuming homogeneous conductivities of 3 S/m for blood and 2 S/m for all other tissues. Forward Matrix were corrected for the Wilson Central Terminal (WCT) reference.

The final dataset contained 680 BSPMs, each associated with one of the 68 corresponding EGMs.

The anatomical torso and atrial meshes used in this study were derived from anonymized MRI-based geometries developed in the works of (Pedrón-Torrecilla et al., 2016) and (Rodrigo et al., 2017). These datasets were generated under institutional ethical approval and include no identifiable patient information. No additional patient data were collected for the present study.

Part of this dataset is publicly available in EDGAR Dataset as Simulation of Atrial Rotors (Valencia_sim_08-01-2014) (Pedrón-Torrecilla et al., 2016; Aras et al., 2015).

### 2.3 Overview of patient groups

The BSPM dataset was developed from simulated EGM signals representing a range of atrial activation patterns. It includes a core (baseline) set of representative rhythms and was further enriched with additional cases to incorporate greater physiological variability and complexity.

The Baseline dataset consists of 520 BSPM recordings generated from 52 unique computational EGM signals (as described in 2.1), each simulating diverse patterns of atrial electrical propagation. This dataset includes two rhythm classes: sinus rhythm and multi-driver AF, corresponding to healthy and pathological conditions, respectively.

To evaluate the model’s ability to generalize beyond the baseline distribution, an extended dataset was introduced, comprising 160 BSPM recordings derived from 16 additional EGM signals. This dataset features less represented activation mechanisms, including ectopic foci originating from both the left atrium (LA) and left pulmonary vein (LPV), as well as AF involving fibrotic substrates. The non-sinus rhythm cases in the dataset differ along two independent factors: (a) the excitation mechanism (e.g., single rotor, multirotor, ectopic activity), and (b) the tissue substrate on which this excitation propagates (presence or absence of fibrosis). In our simulations, all ‘AF multirotor with fibrosis’ cases (Class 5) and some ‘single-rotor’ cases (Class 4) were generated on fibrotic atrial substrates, whereas all remaining classes correspond to non-fibrotic tissue. We include this clarification to make explicit that excitation pattern and substrate condition are conceptually distinct, even though they appear combined in some class labels.

**FIGURE 1:**
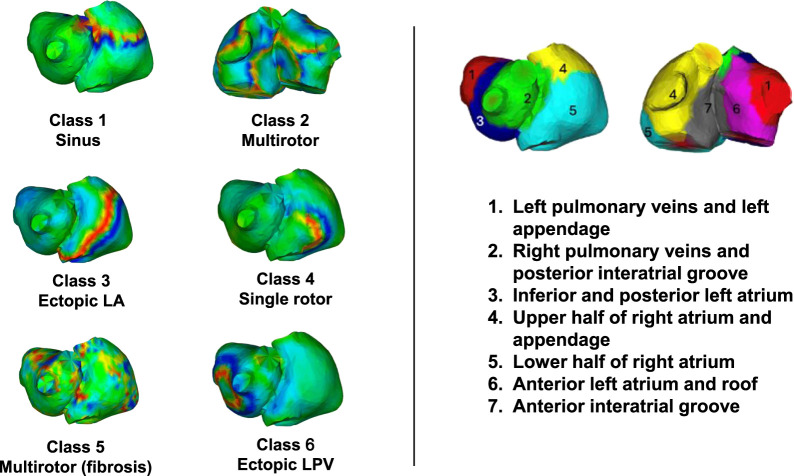
On the left, voltage maps representative of each class are shown. On the right, a schematic of the biatrial anatomy based on a 7-region segmentation of the atria.

In summary, the dataset comprises six classes: Class 1 sinus rhythm, Class 2 multi-rotor atrial fibrillation, Class 3 ectopic origin from the left atrium, Class 4 atrial fibrillation with a single rotor, Class 5 multi-rotor atrial fibrillation with fibrosis, and Class 6 ectopic origin from the left pulmonary veins, as illustrated in Figure 1.

The number of EGMs associated with each class is detailed in Table 1. Together, these classes capture a broad spectrum of electrophysiological behaviors, ranging from organized to highly disorganized atrial dynamics, and from structurally normal to pathologically remodeled substrates.

**TABLE 1:** Number of EGMs per class based on propagation complexity. The Baseline dataset (third column) contains only Classes 1 and 2. The Extended dataset (fourth column) supplements the baseline with additional Classes 3–6, representing less represented activation patterns.

Class ID	Class description	Baseline dataset	Extended dataset
1	Sinus rhythm	19	-
2	Multirotor AF	33	-
3	Ectopic origin - left atrium	-	2
4	One simple rotor	-	4
5	AF multirotor with fibrosis	-	8
6	Ectopic origin - left pulmonary veins	-	2
Number of EGMs		52	16

## 3 Deep learning model architecture and training strategies

This section provides a detailed description of the proposed DL model and the strategies employed to train and evaluate it. We begin by outlining the overall architecture, followed by the preprocessing steps applied to the signal data and their representation for network input. Next, we present the data partitioning, regularization techniques used to improve generalization and robustness and model configuration. Finally, we describe the experimental setup used to assess model performance under different data conditions and training configurations.

### 3.1 Model architecture overview

**FIGURE 2:**
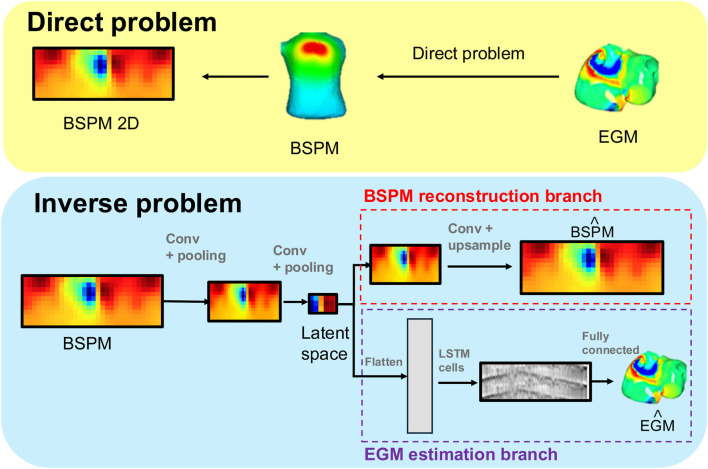
The proposed pipeline includes two main stages: first, the forward problem, where simulated atrial EGMs are used to compute BSPMs using realistic torso models via the direct problem; secondly, the inverse problem, where a DL model estimates EGMs from BSPMs. The dual-branch architecture enables to obtain latent representation of the BSPMs, and leveraging this information, to estimate EGMs.

We implemented a dual-branch neural network architecture to address the estimation problem, as shown in Figure 2. The core component is an encoder that extracts spatiotemporal features from BSPMs and maps them into a compact latent space. From this latent space, two branches operate:

1. BSPM reconstruction branch: a decoder reconstructs the original BSPMs, forcing the latent space to retain sufficient spatiotemporal detail. This encoder-decoder branch effectively acts as a VAE, promoting a smooth and physiologically consistent latent representation.

2. EGM estimation branch: the same latent representation is passed through a set of convolutional layers followed by Long Short-Term Memory (LSTM) blocks, which model temporal dependencies (Hochreiter and Schmidhuber, 1997) to estimate intracardiac EGM signals. This sequence allows the network to capture the evolving electrical activity over each heartbeat and estimate EGM signals from the encoded body-surface data.

In this way, the latent space is shared and enriched by both tasks. The BSPM reconstruction acts as a self-supervised auxiliary task, while the EGM estimation branch represents the main predictive task. This joint training strategy encourages the network to learn a latent representation that captures physiologically relevant dynamics, with the aim of improving generalization in arrhythmic scenarios.

The network was trained to minimize a joint objective based on the VAE framework. We propose a custom loss function for EGM reconstruction, integrating three terms, as defined in Equation 8:

$$L_{\text{total}}=\lambda_{1}\cdot L_{\text{B}}+\lambda_{2}\cdot L_{\text{E}}+L_{\text{K}}$$ (8)

Here, $L_{\text{B}}$ represents the BSPM reconstruction loss, $L_{\text{E}}$ the EGM estimation loss, and $L_{\text{K}}$ the KL-divergence term that regularizes the latent space toward a multivariate Gaussian distribution. All losses are computed as node-wise mean squared error (MSE). The weights $\lambda_{1}=2$ , $\lambda_{2}=8$ were optimized using Optuna (Akiba et al., 2019).

### 3.2 Signal preprocessing and data representation

The preprocessing pipeline transforms raw BSPMs into a format suitable for the network, with two primary goals: (1) to encode the spatial and temporal structure of the signals into a compact image-like representation that preserves topological information, and (2) to enhance physiological realism through signal conditioning and corruption that mimics clinical conditions, as described in the following sub Section 3.2.1 and Section 3.2.2, respectively.

#### 3.2.1 Signal-to-image transformation

The BSPMs represents signals recorded from 64 electrodes distributed across the torso, capturing topological information from different regions. Each BSPM is represented as a matrix of shape N×L , where L=64 corresponds to the number of electrodes (leads) on the torso, and N is the number of time samples, ranging from 1,500 to 4,000 (i.e., 3–8 s at a sampling rate of 500 Hz). The position of the electrodes resemble clinical practice, including frontal, dorsal, and lateral arrangements.

Following the hypothesis that incorporating spatial information is as important as capturing voltage variations over time within a single lead, special attention is given to how wavefronts propagate across neighboring electrodes. With this objective, the BSPM signals were organized into a rectangular array reflecting their spatial layout on the torso. This resuled in an N×6×16 set of images, where N represents the number of temporal samples for each BSPM signal. A further explanation on 2D electrode layout can be found in (Cámara-Vázquez et al., 2021b). To increase the shape of the images and enhance feature extraction inside the network, linear interpolation was applied, obtaining images of size N×16×32 .

#### 3.2.2 BSPM and EGM preprocessing

To simulate real-world conditions and improve model robustness, synthetic corruption was introduced to mimic clinical conditions by adding white noise at 30 SNR to BSPMs. After noise injection, BSPM signals underwent a second 3 − 30 Hz Butterworth band-pass filtering step to attenuate residual noise artifacts.

The BSPMs (and their corresponding EGMs pairs) were then down-sampled to 100 Hz to reduce temporal redundancy and improve computational efficiency during training. This sampling rate was selected to ensure that each data batch retains sufficient temporal resolution to capture complete cardiac cycles, while still aligning with the spectral characteristics of AF, given that the dominant spectral content of AF typically lies below 10 Hz.

Finally, both EGMs and BSPMs are normalized on node-wise to a range between 1 and -1, ensuring consistent magnitude scaling across inputs and reducing the risk of vanishing or exploding gradients during network optimization.

### 3.3 Training and regularization strategies

This subsection describes the procedures used for data partitioning, the regularization techniques implemented to enhance model generalization and robustness and training configuration of parameters.

The baseline dataset and extended dataset, which are comprised on BSPMs-EGMs pairs, is divided into training (80%), validation (5%), and testing (15%) subsets using an EGMs-wise split, ensuring that BSPMs from the same EGM model are not shared across subsets. This prevents data leakage and enables fair evaluation of generalization performance.

To evaluate the model’s robustness and performance, we compared two partitioning approaches in each dataset: a random split and a stratified split, the latter balancing physiological classes (e.g., sinus rhythm vs. AF) across subsets.

After defining the data partitions, we focused on strategies to improve the model’s ability to generalize across rhythm variations. To this end, several regularization techniques were implemented to reduce overfitting and encourage physiologically meaningful representations.

Time masking Inspired by techniques from speech and signal augmentation, time masking involves randomly masking short temporal windows of the input signals during training. These masked segments are replaced with zeros, forcing the model to infer missing information from the surrounding context. This promotes temporal robustness and encourages the network to rely on broader signal structure rather than memorizing exact sequences (Jeong et al., 2021). In this work, masking to zero was applied uniformly across all 64 leads, with random windows of size 40 time steps (20% of batch size) selected per sample.

L2 regularization (weight decay) is incorporated in the optimizer with a coefficient of $1\times 10^{-5}$ , penalizing large weight magnitudes and smoothing the learned function. This helps control model complexity and has been shown effective in improving generalization in deep architectures (Krogh and Hertz, 1992).

Together, these strategies aim to ensure that the model captures physiologically meaningful features that generalize across rhythm classes and anatomical variations, rather than overfitting to dominant patterns like sinus rhythm.

Finally, the training configuration was optimized to promote stable convergence. Training is performed using the Adam optimizer with an initial learning rate of $1\times 10^{-4}$ and a cosine annealing scheduler for gradual decay. The model was trained on 50 epochs, with early stopping applied based on the validation loss with a patience of 15 epochs. A batch size of 100 samples was selected to ensure that each batch contains at least one second of signal, providing sufficient temporal context for learning cardiac dynamics.

The model summary including the layer architecture and number of parameters can be consulted in Supplementary Figures S3 from Supplementary Material.

The experiments were conducted using an NVIDIA GeForce RTX 4090 GPU (24 GB VRAM) with CUDA. Training was performed on a workstation equipped with one RTX 4090 GPU.

### 3.4 Experimental setup

To evaluate the performance and generalization capacity of the proposed model, we conducted experiments in two sequential phases, each designed to test specific aspects of our approach under varying data conditions and training strategies.

#### 3.4.1 Phase 1 - Experiments with baseline dataset

In the first phase, we used the baseline dataset, which includes two physiological classes: multirotor AF and sinus rhythm. These classes represent unique activation patterns: one highly irregular and complex, the other regular and organized. This setting enabled a controlled evaluation of the model’s capacity to estimate both simple and complex rhythms when all classes are well represented during training.

Within this phase, we explored the effect of different training strategies:

- Baseline model performance, without any additional constraints.
- Regularization strategies, including time masking and L2 constraints.
- Stratified sampling with oversampling of class 1 by using simple random oversampling: duplicating samples from the minority classes through sampling with replacement to address class imbalance.

#### 3.4.2 Phase 2 - Experiments with extended dataset

Phase 2 built directly on the best-performing strategies from Phase 1, but expanded the training set by adding four additional, less representated rhythm classes (Classes 3–6: ectopic foci in left atrium/pulmonary veins, single-rotor activity, and AF with fibrosis). The same three training strategies were applied, with stratified oversampling now targeting underrepresented new classes.

This progressive design allowed us to directly observe how training strategies scale with complexity and how well the model architecture generalizes across arrhythmic patterns of varying nature and representation.

## 4 Evaluation

A systematic evaluation framework was accomplished to evaluate and compare the performance of the model in each experiment. The objective is to assess the behaviour of the model under different training conditions, and against the classic benchmark.

### 4.1 Metrics

Evaluation metrics are computed on a node-wise basis, focusing first on two baseline metrics commonly used in ECGI studies: root mean square error (RMSE), which quantifies signal amplitude differences, and the Pearson correlation coefficient, which measures temporal similarity between predicted and ground-truth EGMs.

To provide a more comprehensive characterization of estimation quality, we also included complementary metrics that capture different aspects of signal morphology and spectral fidelity: dynamic time warping (DTW), spectral coherence (SC), and peak detection precision (PDP).

For each experiment, predictions were computed for all test patients. Node-level metrics were computed and then averaged to produce a patient-level score. Group-level results, such as comparisons between sinus rhythm and AF, are reported as mean ± standard deviation across all patients in each rhythm class.

Predictions obtained from the DL model were compared against a classical inverse solution, the zero-order Tikhonov regularization (ZOT), which serves as the classic benchmark reference in this study. Lambda values are optimized for each patient in logaritmic range between $10^{-2}$ and $10^{6}$ using L-curve method. This comparison allows for evaluating whether data-driven approaches can equal or improve traditional model-based techniques under equivalent input conditions.

#### 4.1.1 Root mean square error

Root mean square error (RMSE), defined in Equation 9:

$${\text{RMSE}}_{n}=\sqrt{\frac{1}{T}\overset{T}{\underset{i=1}{\sum }}{\left(y_{i}-{\hat{y}}_{i}\right)}^{2}}$$ (9)

where $y_{i}^{\left(l\right)}$ and ${\hat{y}}_{i}^{\left(l\right)}$ are the ground truth and predicted values at time sample i for node n , and T is the number of time samples. The final RMSE is then obtained as the mean over all nodes N , as defined in Equation 10:

$${\text{RMSE}}_{\text{avg}}=\frac{1}{N}\overset{N}{\underset{n=1}{\sum }}{\text{RMSE}}_{n}$$ (10)

RMSE measures the average magnitude difference between reconstructed and reference signals. While simple and commonly used, RMSE is less physiologically informative on its own. It can be low even in the presence of substantial structural misrepresentations (e.g., flat or phase-inverted signals) (Willmott and Matsuura, 2005).

#### 4.1.2 Pearson correlation

Pearson correlation (Corr), defined in Equation 11:

$$\rho_{n}=\frac{\sum_{i=1}^{T}\left(y_{i}^{\left(n\right)}-{\bar{y}}^{\left(n\right)}\right)\left({\hat{y}}_{i}^{\left(n\right)}-{\bar{\hat{y}}}^{\left(n\right)}\right)}{\sqrt{\sum_{i=1}^{T}{\left(y_{i}^{\left(n\right)}-{\bar{y}}^{\left(n\right)}\right)}^{2}}\sqrt{\sum_{i=1}^{T}{\left({\hat{y}}_{i}^{\left(n\right)}-{\bar{\hat{y}}}^{\left(n\right)}\right)}^{2}}}$$ (11)

where ${\bar{y}}^{\left(n\right)}$ and ${\bar{\hat{y}}}^{\left(n\right)}$ are the mean values of the ground truth and predicted signals for node n , and T is the number of time samples. The final correlation score is the average across all N nodes, as defined in Equation 12:

$$\rho_{\text{avg}}=\frac{1}{N}\overset{N}{\underset{n=1}{\sum }}\rho_{n}$$ (12)

Correlation quantifies the linear similarity between the reconstructed signal and the ground truth. It is highly sensitive to signal shape, but not to absolute magnitude or phase shifts. From a physiological standpoint, a high correlation suggests that the temporal morphology of the activation is preserved, useful for tracking the overall trend of depolarization and repolarization dynamics. However, it can overestimate performance when signals are correctly shaped but misaligned or delayed, which is particularly problematic in arrhythmic scenarios such as multirotor activity (Pearson, 1895).

#### 4.1.3 Dynamic time warping

Dynamic time warping (DTW) is used to quantify the temporal similarity between predicted and ground truth signals, allowing for non-linear temporal alignments. For each node n , the DTW distance between the predicted signal ${\hat{y}}^{\left(n\right)}=\left\{{\hat{y}}_{1}^{\left(n\right)},\ldots ,{\hat{y}}_{N}^{\left(n\right)}\right\}$ and the reference signal $y^{\left(n\right)}=\left\{y_{1}^{\left(n\right)},\ldots ,y_{T}^{\left(n\right)}\right\}$ is computed using the following recursive relation defined in Equation 13:

$$D\left(i,j\right)=\|y_{i}^{\left(n\right)}-{\hat{y}}_{j}^{\left(n\right)}\|+\min\left\{\begin{array}{c} D\left(i-1,j\right),\;\\ D\left(i,j-1\right),\;\\ D\left(i-1,j-1\right)\; \end{array}\right.$$ (13)

with D(0,0)=0 , and the final DTW distance ${\text{DTW}}_{n}$ is given by D(T,T) , the minimal cumulative cost to align both sequences. The average DTW distance across all nodes is given by Equation 14:

$${\text{DTW}}_{\text{avg}}=\frac{1}{N}\overset{N}{\underset{l=1}{\sum }}{\text{DTW}}_{n}$$ (14)

DTW assesses the similarity of signals while allowing for non-linear time deformations. It is particularly valuable in arrhythmic conditions where activation may be delayed, drifted, or exhibit variable cycle lengths. A low DTW distance indicates that reconstructed activation sequences follow the temporal structure of the true signal, even if imperfectly aligned. Physiologically, this supports applications like rotor trajectory reconstruction or estimating phase singularities (Sakoe and Chiba, 2003).

#### 4.1.4 Spectral coherence

Spectral coherence (SC) is calculated between the predicted and true signals, and the maximum coherence value is extracted within a physiologically relevant frequency band (0.5–12 Hz), which captures the dominant spectral content of atrial activity as defined in Equation 15:

$$C_{n}\left(f\right)=\frac{|S_{y^{\left(n\right)}{\hat{y}}^{\left(n\right)}}\left(f\right){|}^{2}}{S_{y^{\left(n\right)}y^{\left(n\right)}}\left(f\right)\cdot S_{{\hat{y}}^{\left(n\right)}{\hat{y}}^{\left(n\right)}}\left(f\right)}$$ (15)

where $S_{y^{\left(n\right)}{\hat{y}}^{\left(n\right)}}\left(f\right)$ is the cross-spectral density between the ground truth and predicted signals, and $S_{y^{\left(n\right)}y^{\left(n\right)}}\left(f\right)$ , $S_{{\hat{y}}^{\left(n\right)}{\hat{y}}^{\left(n\right)}}\left(f\right)$ are their respective power spectral densities.

To summarize coherence performance in a frequency band of interest $\left[f_{\min},f_{\max}\right]$ , the maximum coherence value within the band is extracted for each node, as defined by Equation 16:

$${\text{SC}}_{n}=\max_{f\in \left[f_{\min},f_{\max}\right]}C_{n}\left(f\right)$$ (16)

Finally, the global spectral coherence score is obtained by averaging across all N nodes, as described in Equation 17:

$${\text{SC}}_{\text{avg}}=\frac{1}{N}\overset{N}{\underset{n=1}{\sum }}{\text{SC}}_{n}$$ (17)

Spectral coherence measures the similarity between the frequency components of the reconstructed and ground truth signals, making it particularly relevant for assessing whether dominant rhythms are preserved. Unlike time-domain metrics, it focuses on the alignment of power across frequencies, which is especially informative in AF, where irregular activation may still exhibit consistent dominant frequencies. High spectral coherence values suggest that the estimation captures not just the temporal pattern but also the underlying electrophysiological organization. Furthemore, given its mathematical formulation, this metric is specially sensitive to phase shifts, resulting in low values even in cases where morphology is preserved (Bendat and Piersol, 2011).

#### 4.1.5 Peak detection precision

Peak detection precision is evaluated by identifying local maxima in both the predicted and ground truth signals. A predicted peak is considered correct if it falls within 30% of the expected period relative to its corresponding ground-truth peak, based on the refractory period of atrial tissue.

A predicted peak ${\hat{p}}_{j}\in {\hat{P}}^{\left(n\right)}$ is considered a true positive if there exists a ground truth peak $p_{i}\in P^{\left(n\right)}$ such that the condition in Equation 18 is satisfied:

$$|{\hat{p}}_{j}-p_{i}|\leq \delta_{i}$$ (18)

where $\delta_{i}=0.3\cdot T_{i}$ , and $T_{i}=p_{i}-p_{i-1}$ is the local inter-peak interval (i.e., the estimated physiological period).

The precision for node n is then defined in Equation 19:

$${\text{Precision}}_{n}=\frac{{\text{TP}}_{n}}{{\text{TP}}_{n}+{\text{FP}}_{n}}$$ (19)

where ${\text{TP}}_{n}$ is the number of true positive peaks and ${\text{FP}}_{n}$ the number of unmatched predicted peaks (false positives). The final score is averaged across all N nodes, as defined in Equation 20:

$${\text{Precision}}_{\text{avg}}=\frac{1}{N}\overset{N}{\underset{n=1}{\sum }}{\text{Precision}}_{n}$$ (20)

This metric evaluates the accuracy of detecting activation peaks, which are critical electrophysiological events that correspond to local depolarizations. This metric directly reflects the model’s ability to reconstruct temporal patterns essential for arrhythmia characterization. In cases of AF is a clinically meaningful indicator of morphology fidelity. Correct peak timing is essential for phase mapping, dominant frequency analysis, and rotor tracking.

### 4.2 Qualitative analysis

To assess the physiological relevance of the reconstructed EGMs beyond point-wise accuracy, we generated standard electrophysiological maps from both ground truth and predicted EGMs. These include:

- Voltage maps, computed as the peak-to-peak amplitude over each node’s time series.
- Phase maps, derived using the Hilbert transform applied to the signals, to visualize activation wavefront dynamics.

All maps were projected onto the 3D atrial geometry to enable spatial comparison of reconstructed electrical activity and to identify patterns of regional accuracy or distortion.

## 5 Results

As previously described, the study was structured in two phases. Phase 1 focused exclusively on the baseline dataset, which includes two well-defined rhythm classes: multirotor AF and sinus rhythm. Phase 2 extended the training data by incorporating the extended dataset, which introduced four additional, rarer arrhythmic patterns.

In Phase 1, the stratified model delivered the most consistent and balanced performance, hence it is considered the stratified model. When attending to performance in Class 2 (multirotor), it achieved a correlation of 0.37 ± 0.19, an RMSE of 0.58 ± 0.03, and a peak detection precision (PDP) of 0.71 ± 0.05 (Table 2). These results represent a substantial improvement over the baseline configuration, where correlation dropped to 0.06 ± 0.05 and PDP remained at 0.63 ± 0.06. In contrast, regularization alone yielded minimal gains for Class 2, with correlation only reaching 0.09 ± 0.06.

**TABLE 2:** Performance of the model on Phase **1**. Results are expressed as mean ± standard deviation. CORR: Pearson correlation; RMSE: Root Mean Squared Error; PDP: Peak Detection Precision; DTW: Dynamic Time Warping; COH: Spectral Coherence.

Class	Experiment	CORR	RMSE	PDP	DTW	COH
Class 2 (multi-rotor)	Baseline	0.06 ± 0.05	0.62 ± 0.02	0.63 ± 0.06	204.52 ± 38.44	0.22 ± 0.06
	Stratification	0.37 ± 0.19	0.58 ± 0.03	0.71 ± 0.05	204.52 ± 38.44	0.29 ± 0.06
	Regularization	0.09 ± 0.06	0.61 ± 0.02	0.63 ± 0.06	219.38 ± 46.69	0.22 ± 0.08
Class 1 (sinus rhythm)	Baseline	0.65 ± 0.05	0.43 ± 0.00	0.96 ± 0.00	88.50 ± 0.24	0.91 ± 0.00
	Stratification	0.42 ± 0.16	0.53 ± 0.03	0.90 ± 0.03	93.85 ± 13.76	0.87 ± 0.03
	Regularization	0.66 ± 0.00	0.45 ± 0.00	0.96 ± 0.00	89.09 ± 1.06	0.48 ± 0.01

For Class 1 (sinus rhythm), the model produced the most accurate estimations across all configurations, with correlation above 0.4 in all cases. The stratified model in Phase 1 remained competitive in Class 1 with correlation at 0.42 ± 0.16 and PDP at 0.90 ± 0.03, while delivering notably improved results for the more challenging multirotor signals.

The impact of these differences is highlighted in Table 3, which quantifies interclass performance gaps. The stratified model in Phase 1 had the smallest absolute differences between Class 1 and Class 2 across all metrics: Δ CORR = 0.05, Δ RMSE = 0.05, and Δ PDP = 0.19. These narrow gaps indicate that, when complexity is constrained and class representation is balanced, Phase 1 stratified model generalizes effectively to both simple and complex activation patterns.

**TABLE 3:** Absolute performance difference (indicating by Δ) between Class 1 (Sinus rhythm) and Class 2 (Multirotor) for each experimental configuration. Lower values indicate better generalization across classes. Best performance metrics are highlighted in bold font.

	Experiment	Δ CORR	Δ RMSE	Δ PDP	Δ DTW	Δ COH
Phase 1	Baseline	0.59	0.19	0.33	116.02	0.69
	Stratification	0.05	0.05	0.19	110.67	0.58
	Regularization	0.57	0.16	0.33	130.29	0.26
Phase 2	Baseline	0.56	0.17	0.34	132.00	0.67
	Stratification	0.26	0.10	0.28	159.87	0.67
	Regularization	0.61	0.16	0.33	131.61	0.67

However, when moving to Phase 2, the addition of new arrhythmic classes (ectopic foci, fibrosis) increased the variability and imbalance in the training set. As a result, model performance for Class 2 deteriorated, regardless of the training strategy. Compared to the same model’s results on sinus rhythm, where correlation was 0.46 ± 0.14 and PDP was 0.89 ± 0.12, the widening performance gap is evident: Δ CORR increased to 0.26, and Δ PDP to 0.28 (Table 4).

**TABLE 4:** Performance of the model on Phase **2**. Results are expressed as mean ± standard deviation. CORR: Pearson correlation; RMSE: Root Mean Squared Error; PDP: Peak Detection Precision; DTW: Dynamic Time Warping; COH: Spectral Coherence.

Class	Experiment	CORR	RMSE	PDP	DTW	COH
Class 2 (multi-rotor)	Baseline	0.09 ± 0.16	0.61 ± 0.05	0.61 ± 0.06	221.42 ± 43.76	0.23 ± 0.06
	Stratification	0.20 ± 0.12	0.60 ± 0.01	0.61 ± 0.05	250.82 ± 11.17	0.20 ± 0.02
	Regularization	0.06 ± 0.05	0.60 ± 0.02	0.62 ± 0.04	220.00 ± 47.84	0.22 ± 0.08
Class 1 (sinus rhythm)	Baseline	0.65 ± 0.17	0.44 ± 0.03	0.95 ± 0.02	89.42 ± 17.65	0.90 ± 0.04
	Stratification	0.46 ± 0.14	0.50 ± 0.06	0.89 ± 0.12	90.95 ± 17.13	0.87 ± 0.08
	Regularization	0.67 ± 0.01	0.44 ± 0.10	0.95 ± 0.01	88.39 ± 0.44	0.89 ± 0.01

The degradation was even more severe in the regularized model, where Class 2 correlation dropped to 0.06 ± 0.05, and the interclass correlation gap widened to 0.61. These results suggest that regularization alone fails to mitigate the challenges introduced by increased baseline dataset heterogeneity, especially in underrepresented or electrophysiologically complex classes.

Comparing across phases, it can be noted that under simpler conditions (Phase 1), when guided by a stratified and balanced training strategy, the model was able to estimate both sinus and multirotor signals with high accuracy and consistency as shown by the smallest interclass performance difference. Once class variability increased in Phase 2, this generalization capacity weakened, especially for the more dynamic arrhythmic patterns (Class 1), although stratification continued to offer the most competitive strategy for this class as well.

These quantitative differences were further reflected in the comparison against ZOT, the classical benchmark. To assess whether performance metrics in DL significantly outperform those in ZOT, we employed a paired bootstrap procedure with 5,000 resamples and a significance level of α = 0.1. For each metric, we computed the difference (DL–ZOT) for each of the 12 paired observations (test patients), then repeatedly sampled these differences with replacement to construct the empirical sampling distribution of the mean difference. The 90% confidence interval (CI) of this distribution was used to evaluate significance: if the entire CI lay above zero for metrics where higher values are preferable (Correlation, Peak Detector Precision, Coherence), or below zero for metrics where lower values are preferable (RMSE, DTW), we concluded that DL yielded a statistically significant improvement over ZOT. The complete ZOT results are provided in Supplementary Tables S1, 3 of the Supplementary Material and the results of statistical analysis in Supplementary Table S3.

Overall, the DL model achieved significantly better correlation scores than ZOT in both sinus rhythm and AF cases, indicating a better preservation of temporal morphology. Regarding amplitude error, RMSE values were of similar magnitude for both methods, with ZOT showing slightly lower mean RMSE, although remaining not significant. This behaviour is also visible in Figure 4 for a reference test patient, where the ZOT distribution across nodes exhibits a marginally lower RMSE median despite its poorer correlation. This apparent discrepancy is consistent with the aggregated results: ZOT tends to produce smoother, low-amplitude estimates that reduce RMSE but distort the underlying waveform morphology, while the DL model preserves temporal structure more faithfully at the cost of somewhat higher amplitude error in certain nodes.

### 5.1 EGM analysis

**FIGURE 3:**
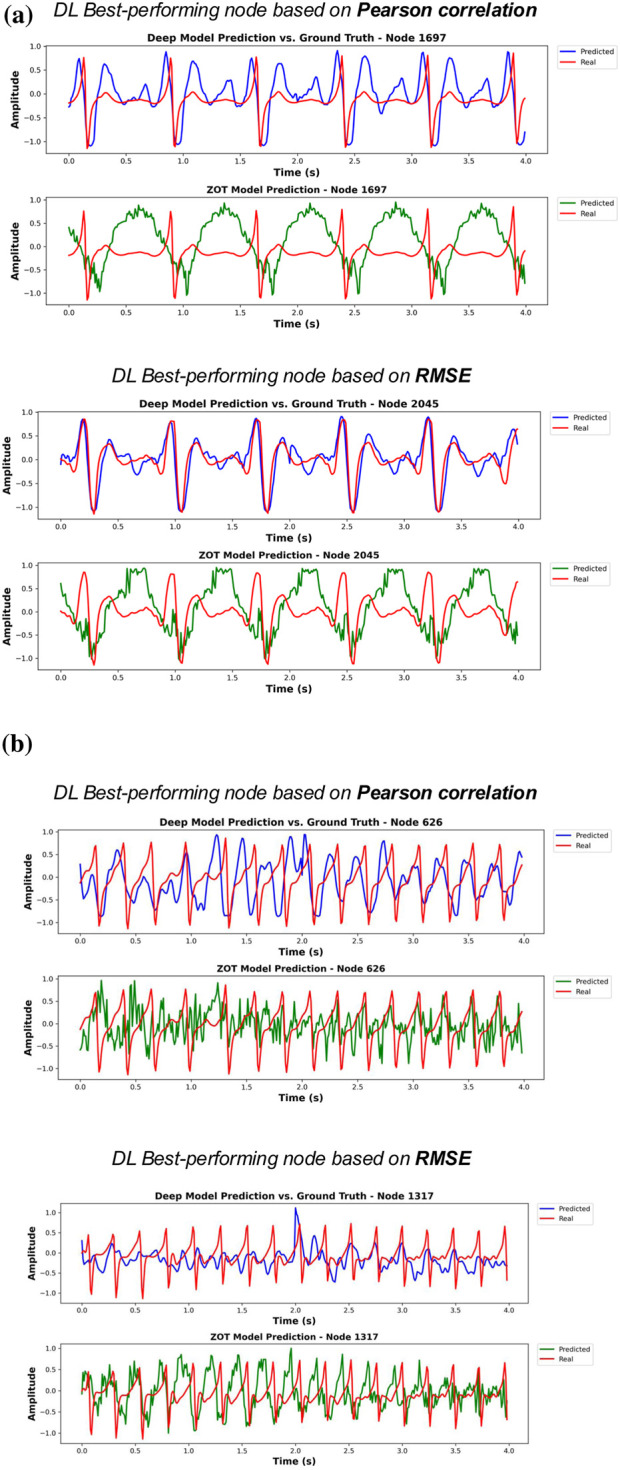
Each figure shows the node with the best DL estimation (best model of Phase 1) performance in terms of correlation (upper panel) and RMSE (lower panel). Subfigure **(a)** presents the optimal results for a patient in sinus rhythm, while subfigure **(b)** shows the best results for AF case.

To visually assess estimation quality, we plot, for each phase, the best-performing node (according to correlation and RMSE) of the best test patient, both for the DL model and for the ZOT solution. Figure 3 and shows the results for Phase 1, Figure 5 presents the corresponding results for Phase 2 and Figure 6 from ZOT reconstruction. Ground truth signals are shown in red, DL estimations in blue, and ZOT reconstructions in green. The same two patients were selected in all cases for consistency: one with sinus rhythm and one with AF due to multirotor activity.

**FIGURE 4:**
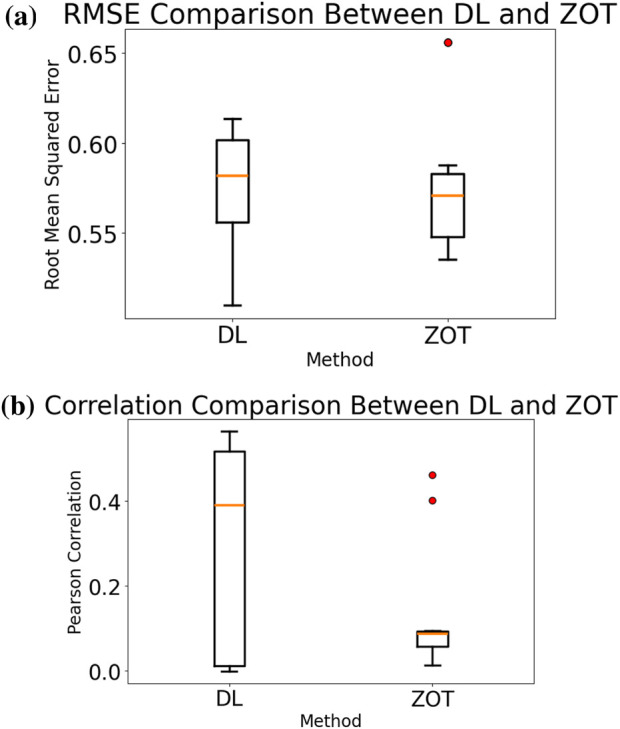
**(a)** RMSE and **(b)** correlation coefficients computed across all atrial nodes for a representative sinus-rhythm patient, comparing the best-performing DL model with the ZOT reconstruction.

**FIGURE 5:**
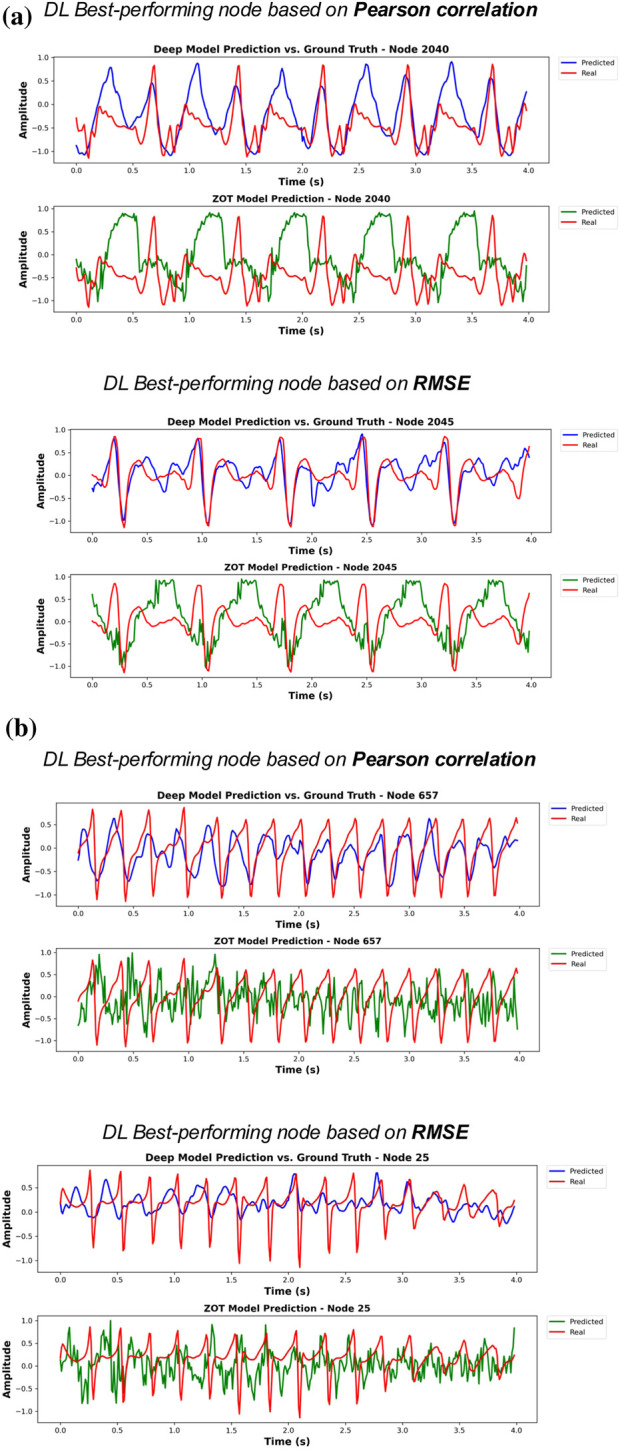
Each figure shows the node with the best DL estimation (best model of Phase 2) performance in terms of correlation (upper panel) and RMSE (lower panel). Subfigure **(a)** presents the optimal results for a patient in sinus rhythm, while subfigure **(b)** shows the best results for AF case.

**FIGURE 6:**
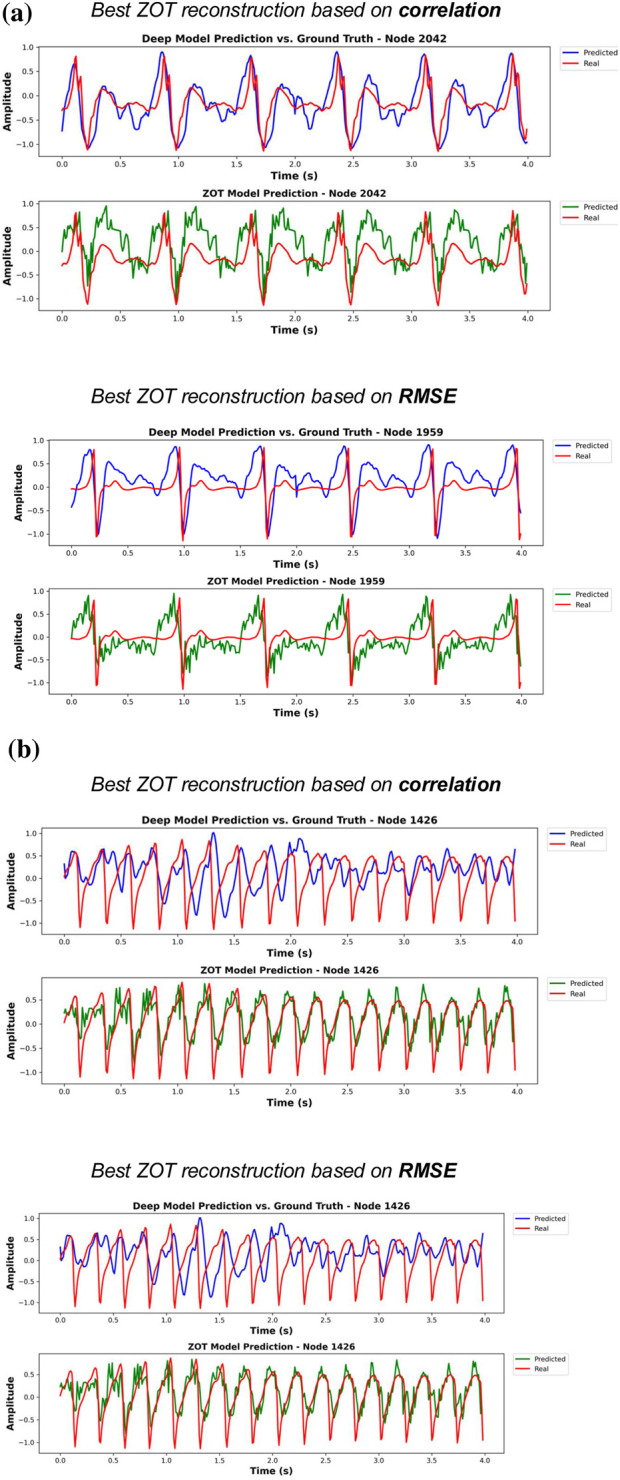
Each figure shows the node with the best ZOT reconstruction performance in terms of correlation (upper panel) and RMSE (lower panel). Subfigure **(a)** presents the optimal results for a patient in sinus rhythm, while subfigure **(b)** shows the best results for AF case.

In general, the stratified model was able to approximate the overall signal morphology in sinus rhythm more consistently than the other configurations, showing sharp deflections and consistent waveforms that closely match the ground truth. Morphological landmarks, such as the P wave and QRS-like deflections, were preserved, although moderate amplitude distortions were observed in some peaks.

For multirotor estimations, the best performing DL model from Phase 1 captured the dominant oscillatory behavior and frequency components, although morphological accuracy was significantly degraded. This was expected due to the complex, irregular nature of AF signals. Notably, in spite of noticeable morphological distortions, the DL estimations were still able to capture elements of the spectral envelope and the dominant periodicity, which is critical for downstream tasks such as dominant frequency mapping or rotor detection. However, in some cases, peaks were temporally misaligned or flattened, especially in low-amplitude regions.

Comparison between phases revealed that the stratified model from Phase 1 yielded better qualitative results, particularly in rhythm regularity for AF cases and waveform morphology for sinus rhythm. This aligns with the spectral coherence findings (0.29 in Phase 1 vs. 0.20 in Phase 2) and suggests that lower interclass variability improves learning of temporal dynamics. In contrast, the stratified model from Phase 2, displayed greater variability in amplitude and frequency content.

Importantly, ZOT reconstructions, while often preserving the rhythm and general periodicity, suffered from significant morphological artifacts, such as spurious high or low-frequency components, which distorted the signal envelope. This is likely due to the lack of regularization from a general lambda tuning and absence of post-processing in this setup. DL-based methods, even without post-processing of predictions, yielded more physiologically coherent estimations.

**FIGURE 7:**
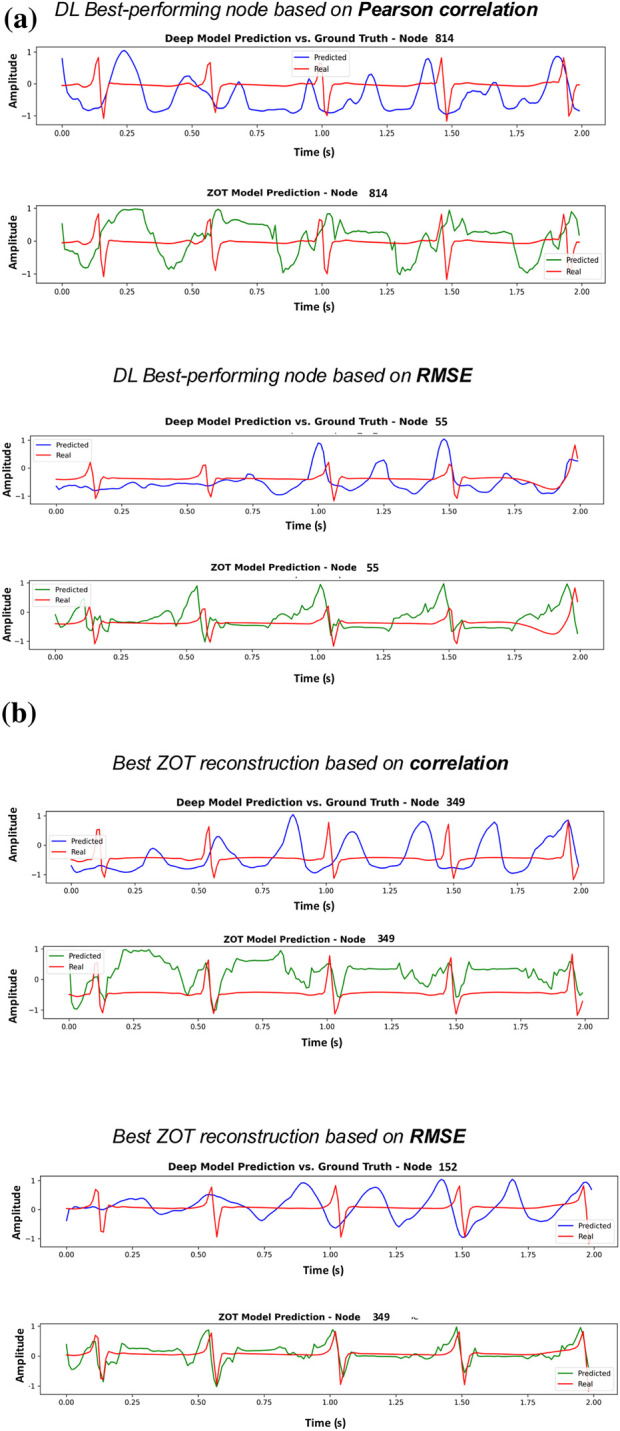
Results for the worst-performing test patient in terms of correlation (best model from Phase 1). Each subfigure displays the node with the highest correlation (top panel) and the node with the lowest RMSE (bottom panel). Subfigure **(a)** shows the corresponding DL best-performing node estimations, while subfigure **(b)** shows the ZOT best-performing node reconstructions for the same patient.

Furthermore, the worst-performing patient for the DL model is shown in Figure 7, and notably, this same patient corresponds to the best-performing case for ZOT in terms of correlation. As reflected in the quantitative metrics, the DL estimations for this patient are not satisfactory, whereas ZOT achieves comparatively good performance. We hypothesize that this discrepancy may be related to the characteristics of the underlying EGM signal, which exhibits a markedly different visual appearance compared with the rest of the dataset representative samples for each EGM are provided in the Supplementary Material). Such atypical patterns are likely underrepresented in the training set, causing the DL model to generalize poorly in this scenario—an issue that does not affect ZOT, whose performance does not depend on data-driven training.

Overall, this analysis reinforces the central observation from the quantitative results: models trained in simplified, balanced settings (Phase 1) consistently produced cleaner and more interpretable estimations, even for arrhythmic signals. In contrast, increased training complexity in Phase 2 reduced morphological similarity, particularly in cases with rich temporal dynamics. These findings emphasize the importance of data stratification and complexity-aware training design in non-invasive EGM estimation using DL, where interpretability and robustness of EGM morphology are critical for downstream tasks such as rotor localization and phase mapping.

### 5.2 Analysis of spatial behaviour

While the current analysis focuses on single-node estimations to highlight signal-level accuracy, EGM morphology alone does not capture the full picture of activation behavior. To address this, this section evaluates the spatial coherence of the estimations across the full atrial surface using 3D maps of voltage and phase dynamics. Specifically, we analyzed voltage and phase maps generated from the predicted EGMs.

**FIGURE 8:**
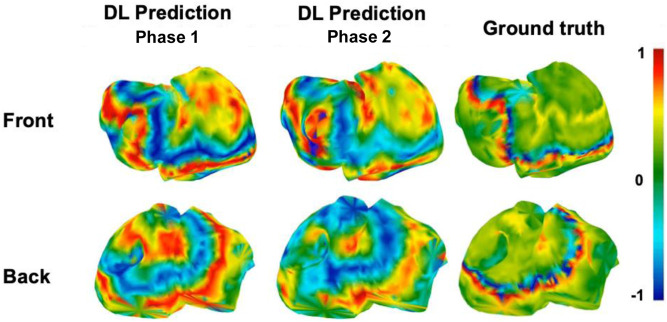
Voltage map example from sinus patient for the case for best DL model in Phase 1 and Phase 2

**FIGURE 9:**
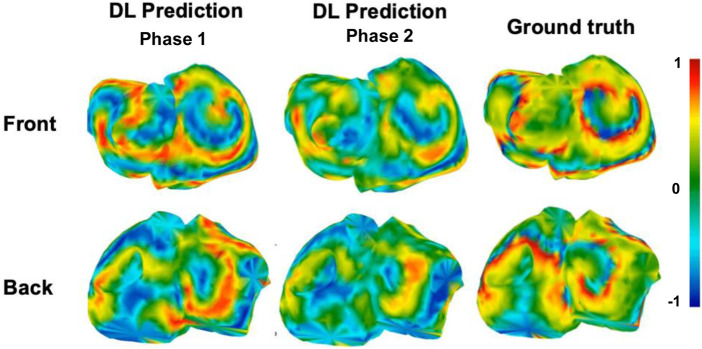
Voltage map example from AF patient for the case for best DL model in Phase 1 and Phase 2

**FIGURE 10:**
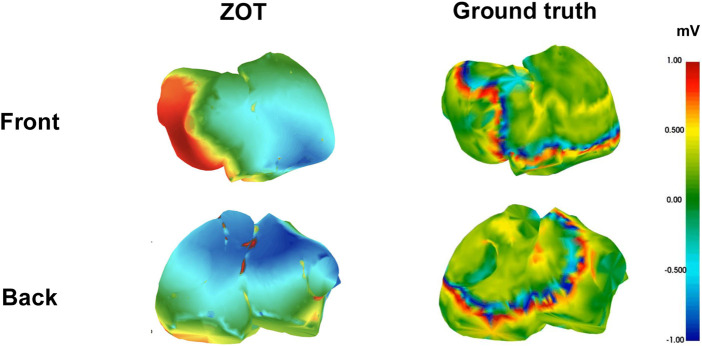
Voltage map example from sinus patient reconstructed using ZOT.

Figures 8, 9 show representative voltage maps at a fixed time point for both sinus rhythm and multi-rotor cases, comparing predictions from the DL model trained under Phase 1 and Phase 2 settings with the ground truth. Figure 10 represents an estimation using ZOT of the same sinus patient, for reference.

From the front and back views, the best performing model from Phase 1 exhibits a closer qualitative resemblance to the ground truth, particularly in the general continuity and polarity of wavefronts across both atrial surfaces. It reproduces broad activation gradients and some of the higher-amplitude regions with greater consistency, suggesting that the global organization of the voltage field is partially retained. In contrast, the stratified model from Phase 2 shows more fragmentation and spatial smoothing, especially in areas with rapid voltage transitions. Nonetheless, noticeable discrepancies in peak-amplitude regions (e.g., exaggerated red/yellow patches) highlight persistent limitations in fine-scale voltage estimation, which may influence downstream applications such as rotor localization or substrate assessment, consistent with the waveform-level observations in Figure 3.

**FIGURE 11:**
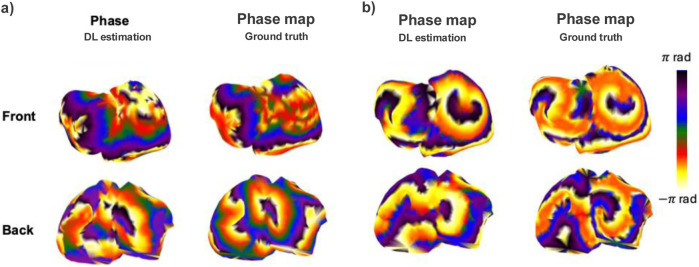
Phase mapping results from the Phase 1 stratified model. The left panel **(a)** shows phase maps for a sinus rhythm patient, and the right panel **(b)** shows phase maps for an AF patient. For each case, the left column presents the deep learning (DL) estimation, and the right column shows the ground truth. The first row corresponds to the front atrial view, and the second row to the back atrial view. Phase values are color-coded from −π to π radians.

To further assess the spatiotemporal coherence of the reconstructed signals, we analyzed phase maps derived from the predicted EGMs using Hilbert transform-based method (Figure 11). These maps were computed only for the best performing model, Phase 1 model, and considering two patients: sinus and multirotor patient. For sinus rhythm (panel a), the Phase 1 model successfully reproduced the expected smooth propagation of activation, with consistent phase gradients and directionality across both atrial surfaces. Minor discrepancies were confined to regions of steep phase transitions, likely due to slight waveform shape differences rather than gross structural errors. In the AF scenario (panel b), the Phase 1 estimation preserved key dynamic features, including rotational activity and disorganized, reentrant wavefronts. Although the exact location and sharpness of phase singularities showed some deviation from the ground truth, the model retained the global structure of chaotic propagation.

**FIGURE 12:**
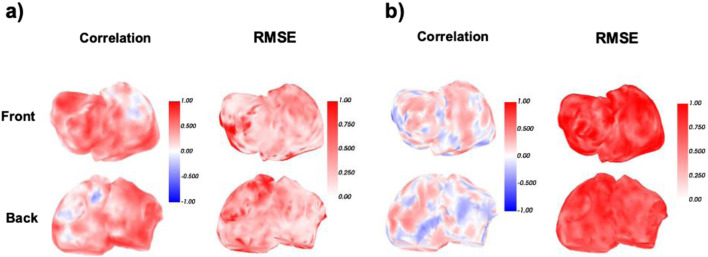
Spatial distribution of correlation (left) and normalized RMSE (right) between DL predicted and ground truth EGMs, for representative cases of **(a)** sinus rhythm and **(b)** AF (AF) with multirotor dynamics. All maps correspond to the model trained using Phase 1. In sinus rhythm, both metrics indicate high spatial accuracy across the atrial surface. In AF, the model maintains moderate to high correlation in several regions and captures global signal structure despite the increased spatiotemporal complexity, highlighting its capacity to generalize across arrhythmic conditions.

Complementing this analysis, Figure 12 shows the spatial distribution of correlation and RMSE between predicted and ground truth EGMs for the same two patients, based on the Phase 1: (a) sinus rhythm and (b) AF with multirotor dynamics. Both maps correspond to the model trained in Phase 1.

In the sinus rhythm case, correlation values are uniformly high across the entire atrial surface, with most regions exceeding 0.75, indicating excellent temporal reconstruction between predicted and reference signals. RMSE is also consistently low, reflecting precise voltage amplitude estimation and confirming the model’s strong performance. In the AF case, although the correlation map reveals greater spatial variability, particularly in posterior regions, the model still captures key activation patterns with moderate to high correlation in many areas. Notably, several atrial zones maintain correlation values above 0.5 despite the intrinsic irregularity of multirotor propagation.

## 6 Conclusion

This study presents a DL-based model using VAEs to estimate atrial EGMs from BSPMs. Results demonstrate that the composition and distribution of training data strongly influence model performance and generalization for non-invasive EGM estimation task. When trained exclusively on the main dataset (Phase 1), which includes two well-represented physiological classes, the method captured coarse features of the activation dynamics, but accurate waveform reconstruction, especially in AF, remains a challenge. However, when additional arrhythmic classes from the extended dataset were introduced in Phase 2, representing more heterogeneous propagation mechanisms, the model’s generalization performance declined, particularly for underrepresented patterns.

The results demonstrate that, when trained with physiologically stratified data, the proposed method is capable of partially reconstructing intracardiac signals and preserving some temporal and spectral trends. Importantly, this includes the reconstruction of activation peaks, spectral coherence, and voltage gradients, which are critical for identifying arrhythmogenic substrates such as rotors, focal sources, or fibrotic areas. Compared to traditional inverse methods like ZOT, the DL model achieved superior morphological fidelity and temporal coherence, especially in cases of complex AF dynamics.

From a clinical perspective, these findings support the feasibility of non-invasive, data-driven functional mapping and point toward its potential future role in personalizing ablation strategies. The ability to estimate EGMs with physiologically plausible morphology and spatial coherence directly from BSPMs may reduce reliance on prolonged and invasive intracardiac mapping procedures, facilitating earlier and safer identification of ablation targets. This method is not meant to override invasive mapping, but rather to complement it by providing global insights derived from non-invasive estimation.

Nonetheless, as a key cornerstone coming from this study is the critical need for diverse and physiologically representative datasets. While this work addresses the non-invasive EGM estimation problem within a controlled, simulation-based environment, it inherently faces limitations related to dataset diversity and scale. To enable clinical translation and broader applicability, it is essential to develop high-quality, well-stratified datasets that capture the full spectrum of atrial electrophysiological behavior. Only through such representative data can DL models be trained to robustly estimate complex electrical patterns and generalize effectively across patient populations with variable anatomical and pathological conditions. However, categorizing arrhythmic signals solely on the basis of visual dynamics is imprecise and subject to substantial inter-operator variability. Future work should investigate stratification strategies grounded in established clinical classifications (e.g., persistent AF and other ISO-defined subtypes). VAEs may further aid in disentangling distinct rhythm patterns that coexist within the same disease category. In addition, incorporating more advanced monodomain electrophysiological models represents an important direction for future work, as it would provide higher-quality and more physiologically accurate simulated data. Likewise, expanding the forward-model framework to multiple realistic atrial anatomies, including population-derived or generic meshes, will allow to train and evaluate the model across anatomical variability.

While this study demonstrates the potential of deep learning models, particularly VAEs, in addressing the electrocardiographic inverse problem, several limitations and opportunities for future research remain. First, the present work focuses on the intermediate task of non-invasive EGM reconstruction rather than direct AF substrate identification. This choice reflects the lack of reliable functional ground truth in AF, which prevents data-driven models from being trained or validated directly on substrate-level targets such as rotors, focal sources, or low-voltage areas. Consequently, reconstructed EGMs serve as a necessary foundation for future substrate-based mapping rather than an end point by themselves.

Second, although the dataset used in this study is physiologically diverse, it is derived from simulated atrial models and a limited number of torso geometries. The mThis may not fully capture the anatomical variability, noise sources, and electrode placement inconsistencies found in clinical practice. When additional arrhythmic mechanisms were incorporated, model performance declined, revealing sensitivity to class imbalance and highlighting the importance of larger and more representative datasets. Alternative strategies to increase dataset diversity, such as advanced data augmentation, generative models (VAEs or diffusion models), or synthetic minority oversampling, should be explored to better cover underrepresented electrophysiological patterns.

Third, although simulations were used for both training and evaluation, several safeguards were implemented to avoid inverse crime, including variation of torso geometries, noise corruption, and patient-level partitioning. Nonetheless, future extensions should incorporate forward models with heterogeneous meshes, conductivities, anatomical variability and electrode disconnection to further test robustness.

Finally, future work may benefit from exploring the use of large-scale foundation models as pretraining backbones or initialization strategies, which could enhance generalization across diverse anatomical and electrophysiological conditions. Overall, the proposed method should be viewed as a complementary tool, not a replacement, for invasive mapping, providing global non-invasive information that could support or streamline clinical workflows. Importantly, future studies must determine whether such reconstructions improve correspondence with ablation outcomes, rather than assuming that increased spatial resolution is intrinsically beneficial. This is particularly relevant in AF, where functional markers such as dominant frequency, voltage, or phase singularities are highly sensitive to sampling density and may benefit more from coherent global reconstruction than from fine structural detail. Ultimately, clinical validation on patient-specific datasets will be essential to fully assess the translational potential of this approach.

## Data Availability

The data analyzed in this study is subject to the following licenses/restrictions: Access to the dataset is restricted due to ownership by a collaborating research group and confidentiality agreements. Requests to access these datasets should be directed to mguisan@eln.upv.es.

## References

[B1] Akiba T. Sano S. Yanase T. Ohta T. Koyama M. (2019). “Optuna: a next-generation hyperparameter optimization framework,” in Proceedings of the 25th ACM SIGKDD International Conference on Knowledge Discovery and Data Mining (ACM). 10.1145/3292500.3330701

[B2] Aras K. Good W. Tate J. Burton B. Brooks D. Coll-Font J. (2015). Experimental data and geometric analysis repository (edgar). J. Electrocardiol. 48, 975–981. 10.1016/j.jelectrocard.2015.08.008 26320369 PMC4624576

[B3] Atienza F. Almendral J. Jalife J. Zlochiver S. Ploutz-Snyder R. Torrecilla E. G. (2009). Real-time dominant frequency mapping and ablation of dominant frequency sites in atrial fibrillation with left-to-right frequency gradients predicts long-term maintenance of sinus rhythm. Heart Rhythm. 6, 33–40. 10.1016/j.hrthm.2008.10.024 19121797 PMC2867332

[B4] Bacoyannis T. Ly B. Cochet H. Sermesant M. (2022). Deep learning formulation of ECGI evaluated on clinical data. EP Eur. 24, euac053.566. 10.1093/europace/euac053.566

[B5] Bailin S. J. Korthas M. A. Weers N. J. Hoffman C. J. (2011). Direct visualization of the slow pathway using voltage gradient mapping: a novel approach for successful ablation of atrioventricular nodal reentry tachycardia. Europace 13, 1188–1194. 10.1093/europace/eur112 21508003

[B6] Bendat J. S. Piersol A. G. (2011). Random data: analysis and measurement procedures. John Wiley and Sons.

[B7] Cámara-Vázquez M. Á. Hernández-Romero I. Rodrigo M. Alonso-Atienza F. Figuera C. Morgado-Reyes E. (2021a). Electrocardiographic imaging including intracardiac information to achieve accurate global mapping during atrial fibrillation. Biomed. Signal Process. Control 64, 102354. 10.1016/j.bspc.2020.102354

[B8] Cámara-Vázquez M. Á. Hernández-Romero I. Morgado E. Guillem M. S. Climent A. M. Barquero-Pérez Ó. (2021b). Non-invasive estimation of atrial fibrillation driver position with convolutional neural networks and body surface potentials. Front. Physiology 12, 733449. 10.3389/fphys.2021.733449 PMC8552066 34721065

[B9] Chen K. W. Bear L. Lin C. W. (2022). Solving inverse electrocardiographic mapping using machine learning and deep learning frameworks. Sensors 22, 2331. 10.3390/s22062331 35336502 PMC8951148

[B10] Cheniti G. Puyo S. Martin C. A. Frontera A. Vlachos K. Takigawa M. (2019). Noninvasive mapping and electrocardiographic imaging in atrial and ventricular arrhythmias (cardioinsight). Card. Electrophysiology Clinics 11, 459–471. 10.1016/j.ccep.2019.05.004 31400870

[B11] Chira D. Haralampiev I. Winther O. Dittadi A. Liévin V. (2022). “Image super-resolution with deep variational autoencoders,” in European Conference on Computer Vision (Springer), 395–411.

[B12] Cuculich P. S. Wang Y. Lindsay B. D. Faddis M. N. Schuessler R. B. Damiano Jr R. J. (2010). Noninvasive characterization of epicardial activation in humans with diverse atrial fibrillation patterns. Circulation 122, 1364–1372. 10.1161/CIRCULATIONAHA.110.945709 20855661 PMC2996091

[B13] Elliott A. D. Middeldorp M. E. Van Gelder I. C. Albert C. M. Sanders P. (2023). Epidemiology and modifiable risk factors for atrial fibrillation. Nat. Rev. Cardiol. 20, 404–417. 10.1038/s41569-022-00820-8 36600003

[B14] Figuera C. Suárez-Gutiérrez V. Hernández-Romero I. Rodrigo M. Liberos A. Atienza F. (2016). Regularization techniques for ecg imaging during atrial fibrillation: a computational study. Front. Physiology 7, 466. 10.3389/fphys.2016.00466 27790158 PMC5064166

[B15] Goh H. Sheriffdeen S. Wittmer J. Bui-Thanh T. (2019). Solving bayesian inverse problems via variational autoencoders.

[B16] Goldberger J. J. (2017). Substrate ablation for treatment of atrial fibrillation: back to basics. J. Cardiovasc. Electrophysiol. 28, 156–158. 10.1111/jce.13149 27957770

[B17] Gómez-Doblasa J. J. López-Garrido M. A. Esteve-Ruiz I. Barón-Esquivias G. (2016). Epidemiología de la fibrilación auricular. Rev. Esp. Cardiol. Supl. 16A, 2–7. 10.1016/S1131-3587(16)30007-3

[B18] Gutiérrez-Fernández-Calvillo M. Cámara-Vázquez M. Á. Hernández-Romero I. Guillem M. S. Climent A. M. Fambuena-Santos C. (2024). Non-invasive estimation of atrial fibrillation driver position using long-short term memory neural networks and body surface potentials. Comput. Methods Programs Biomed. 246, 108052. 10.1016/j.cmpb.2024.108052 38350188

[B19] Gutiérrez-Fernández M. Cámara-Vázquez M. A. Hernández-Romero I. Fambuena-Santos C. Guillem M. S. Climent A. M. (2023). “Egm reconstruction from bsps in atrial fibrillation using deep learning,” in 2023 Computing in Cardiology (CinC), 1–4. 10.22489/CinC.2023.302

[B20] Haissaguerre M. Hocini M. Denis A. Shah A. J. Komatsu Y. Yamashita S. (2014). Driver domains in persistent atrial fibrillation. Circulation 130, 530–538. 10.1161/CIRCULATIONAHA.113.005421 25028391

[B21] Hernández-Romero I. Molero R. Fambuena-Santos C. Herrero-Martin C. Climent A. M. Guillem M. S. (2023). Electrocardiographic imaging in the atria. Med. and Biol. Eng. and Comput. 61, 879–896. 10.1007/s11517-022-02709-7 36370321 PMC9988819

[B22] Heydari A. A. Mehmood A. (2020). Srvae: super resolution using variational autoencoders. *Pattern Recognit. and Track. XXXI* (SPIE) 11400, 87–100. 10.1117/12.2559808

[B23] Hochreiter S. Schmidhuber J. (1997). Long short-term memory. Neural Comput. 9, 1735–1780. 10.1162/neco.1997.9.8.1735 9377276

[B24] Jeong C. Y. Shin H. C. Kim M. (2021). Sensor-data augmentation for human activity recognition with time-warping and data masking. Multimedia Tools Appl. 80, 20991–21009. 10.1007/s11042-021-10600-0

[B25] Knackstedt C. Schauerte P. Kirchhof P. (2008). Electro-anatomic mapping systems in arrhythmias. Europace 10, iii28–iii34. 10.1093/europace/eun225 18955396

[B26] Krogh A. Hertz J. A. (1992). A simple weight decay can improve generalization. Adv. Neural Inf. Process. Syst. 4, 950–957.

[B27] La R. G. Quintanilla J. G. Salgado R. González-Ferrer J. J. Cañadas-Godoy V. Pérez-Villacastín J. (2021). Anatomical targets and expected outcomes of catheter-based ablation of atrial fibrillation in 2020. Pacing Clin. Electrophysiol. 44, 341–359. 10.1111/pace.14140 33283883

[B28] Lacki A. Hernández-Romero I. Guillem M. S. Climent A. M. (2021). “ECGI periodicity unraveled: a deep learning approach for the visualization of periodic spatiotemporal patterns in atrial fibrillation patients,” in 2021 Computing in Cardiology (CinC) (Czech Republic: IEEE), 1–4.

[B29] Lawson C. L. (1984). C^1^ surface interpolation for scattered data on a sphere. Rocky Mt. J. Math. 177–202.

[B30] Pappone C. Santinelli V. (2006). Substrate ablation in treatment of atrial fibrillation. J. Cardiovasc. Electrophysiol. 17, S23–S27. 10.1111/j.1540-8167.2006.00630.x

[B31] Pearson K. (1895). Vii. note on regression and inheritance in the case of two parents. Proc. R. Soc. Lond. 58, 240–242. 10.1098/rspl.1895.0041

[B32] Pedrón-Torrecilla J. Rodrigo M. Climent A. M. Liberos A. Pérez-David E. Bermejo J. (2016). Noninvasive estimation of epicardial dominant high-frequency regions during atrial fibrillation. J. Cardiovasc. Electrophysiol. 27, 435–442. 10.1111/jce.12931 26776725 PMC5547887

[B33] Prost J. Houdard A. Almansa A. Papadakis N. (2023). “Inverse problem regularization with hierarchical variational autoencoders,” in Proceedings of the IEEE/CVF International Conference on Computer Vision, 22894–22905.

[B34] Rodrigo M. Climent A. M. Hernández-Romero I. Liberos A. Baykaner T. Rogers A. J. (2020). Noninvasive assessment of complexity of atrial fibrillation: correlation with contact mapping and impact of ablation. Circulation Arrhythmia Electrophysiol. 13, e007700. 10.1161/CIRCEP.119.007700 32078374 PMC7508259

[B35] Rodrigo M. Climent A. M. Liberos A. Fernández-Avilés F. Berenfeld O. Atienza F. (2017). Technical considerations on phase mapping for identification of atrial reentrant activity in direct-and inverse-computed electrograms. Circulation Arrhythmia Electrophysiol. 10, e005008. 10.1161/CIRCEP.117.005008 28887361

[B36] Rudy Y. Burnes J. E. (1999). Noninvasive electrocardiographic imaging. Ann. Noninvasive Electrocardiology 4, 340–359. 10.1111/j.1542-474x.1999.tb00220.x

[B37] Sakoe H. Chiba S. (2003). Dynamic programming algorithm optimization for spoken word recognition. IEEE Trans. Acoust. Speech, Signal Process. 26, 43–49. 10.1109/tassp.1978.1163055

[B38] Salinet J. Molero R. Schlindwein F. S. Karel J. Rodrigo M. Rojo-Álvarez J. L. (2021). Electrocardiographic imaging for atrial fibrillation: a perspective from computer models and animal experiments to clinical value. Front. Physiology 12, 653013. 10.3389/fphys.2021.653013 33995122 PMC8120164

[B39] Tenderini R. Pagani S. Quarteroni A. Deparis S. (2022). Pde-aware deep learning for inverse problems in cardiac electrophysiology. SIAM J. Sci. Comput. 44, B605–B639. 10.1137/21m1438529

[B40] Tsyganov A. Petru J. Skoda J. Sediva L. Hala P. Weichet J. (2015). Anatomical predictors for successful pulmonary vein isolation using balloon-based technologies in atrial fibrillation. J. Interventional Cardiac Electrophysiol. 44, 265–271. 10.1007/s10840-015-0068-3 26475792

[B41] Van Gelder I. C. Rienstra M. Bunting K. V. Casado-Arroyo R. Caso V. Crijns H. J. G. M. (2024). ESC guidelines for the management of atrial fibrillation developed in collaboration with the european association for cardio-thoracic surgery (EACTS). Eur. Heart J. 45, 3314–3414. 10.1093/eurheartj/ehae176 39210723

[B42] Wang T. Karel J. M. Osnabrugge N. Driessens K. Stoks J. Cluitmans M. J. (2025). Deep learning based estimation of heart surface potentials. Artif. Intell. Med. 163, 103093. 10.1016/j.artmed.2025.103093 40073713

[B43] Willmott C. J. Matsuura K. (2005). Advantages of the mean absolute error (mae) over the root mean square error (rmse) in assessing average model performance. Clim. Res. 30, 79–82. 10.3354/cr030079

[B44] Xie J. Yao B. (2022). Physics-constrained deep learning for robust inverse ecg modeling. IEEE Trans. Automation Sci. Eng. 20, 151–166. 10.1109/tase.2022.3144347

[B45] Xiao H. Yang Z. Liu T. Liu S. Huang X. Dai J. (2025). Deep learning for medical imaging super-resolution: a comprehensive review. Neurocomputing 630, 129667. 10.1016/j.neucom.2025.129667

[B46] Zheng Y. Xia Y. Carlson J. Kongstad O. Yuan S. (2017). Atrial average conduction velocity in patients with and without paroxysmal atrial fibrillation. Clin. Physiology Funct. Imaging 37. 596–601. 10.1111/cpf.12342 26762841

