# Correction: Deep learning for atrial electrogram estimation: toward non-invasive arrhythmia mapping using variational autoencoders

**DOI:** 10.3389/fphys.2026.1788430

**Published:** 2026-01-26

**Authors:** Miriam Gutiérrez-Fernández, K. López-Linares, C. Fambuena-Santos, Maria S. Guillem, Andreu M. Climent, Ó. Barquero-Pérez

**Affiliations:** 1 Signal Theory and Communications Dpt., EIF, Universidad Rey Juan Carlos, Fuenlabrada, Spain; 2 Vicomtech Foundation, Basque Research and Technology Alliance (BRTA), San Sebastián, Spain; 3 eHealth Group, Bioengineering Area, Biogipuzkoa Health Research Institute, San Sebastián, Spain; 4 ITACA Institute, Universitat Politècnica de València, València, Spain

**Keywords:** atrial fibrillation, body surface potential mapping, deep learning, inverse problem, variational autoencoder

In the published article, there was an error in the author **Affiliations**.

This correction addresses administrative errors in the published **Affiliations** and does not affect the scientific content of the article. **Affiliations** 2 and 3 were published incorrectly in the original article. The correct **Affiliations** are:

2Vicomtech Foundation, Basque Research and Technology Alliance (BRTA), San Sebastián, Spain

3eHealth Group, Bioengineering Area, Biogipuzkoa Health Research Institute, San Sebastián, Spain

The original article has been updated.

