# Supplementary figures and images for: Deep learning for atrial electrogram estimation: toward non-invasive arrhythmia mapping using variational autoencoders
Source: Front Physiol. 2026 Jan 12;16:1720244. doi: 10.3389/fphys.2025.1720244 (PMC12832759; doi:10.3389/fphys.2025.1720244)

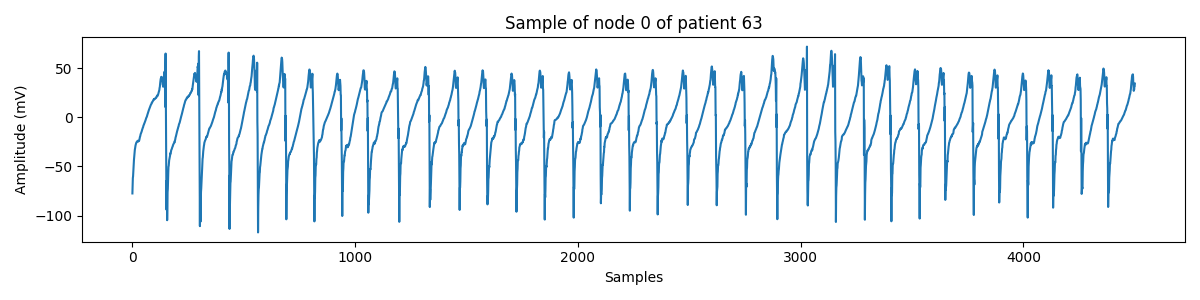

Supplement: Supplementary file 1 [file DataSheet1.zip › EGMs_sample/63.png]

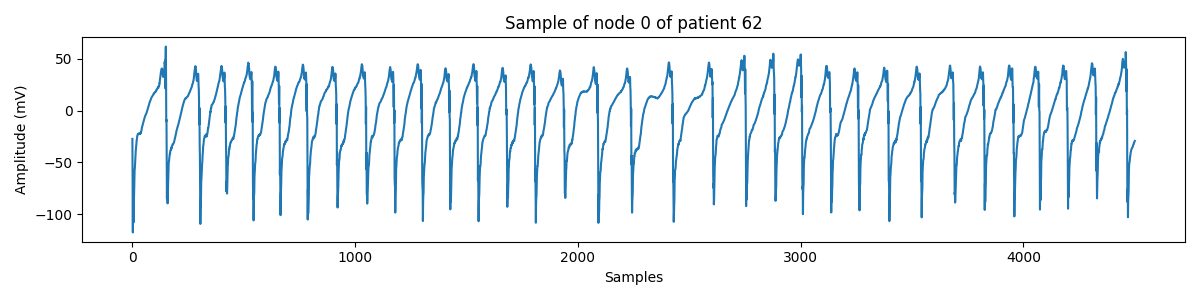

Supplement: Supplementary file 1 [file DataSheet1.zip › EGMs_sample/62.png]

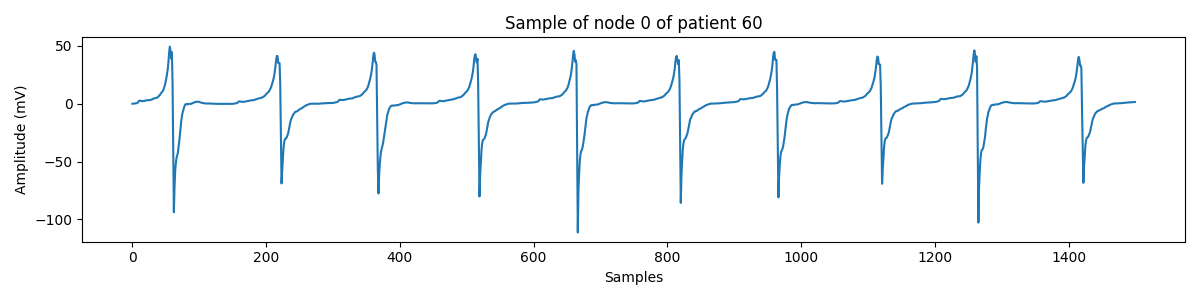

Supplement: Supplementary file 1 [file DataSheet1.zip › EGMs_sample/60.png]

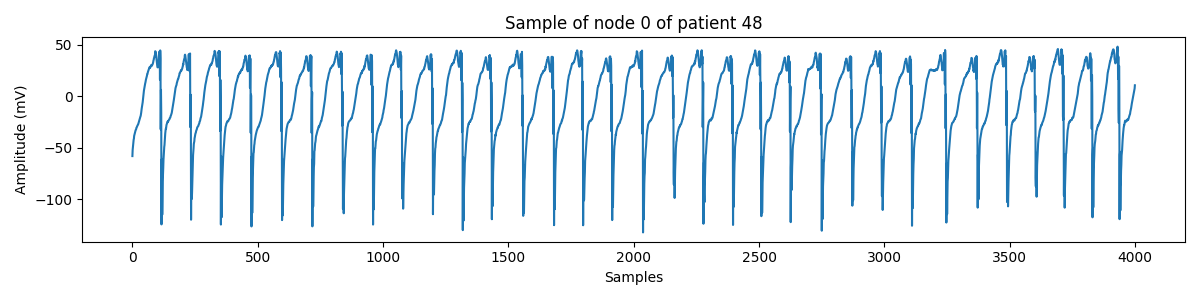

Supplement: Supplementary file 1 [file DataSheet1.zip › EGMs_sample/48.png]

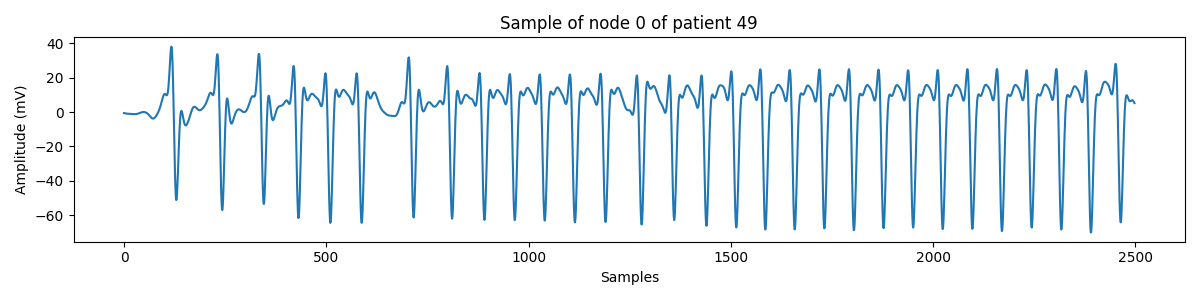

Supplement: Supplementary file 1 [file DataSheet1.zip › EGMs_sample/49.png]

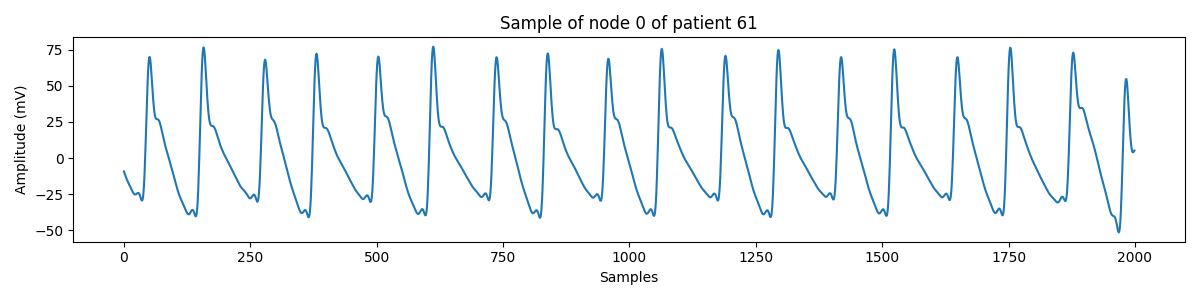

Supplement: Supplementary file 1 [file DataSheet1.zip › EGMs_sample/61.png]

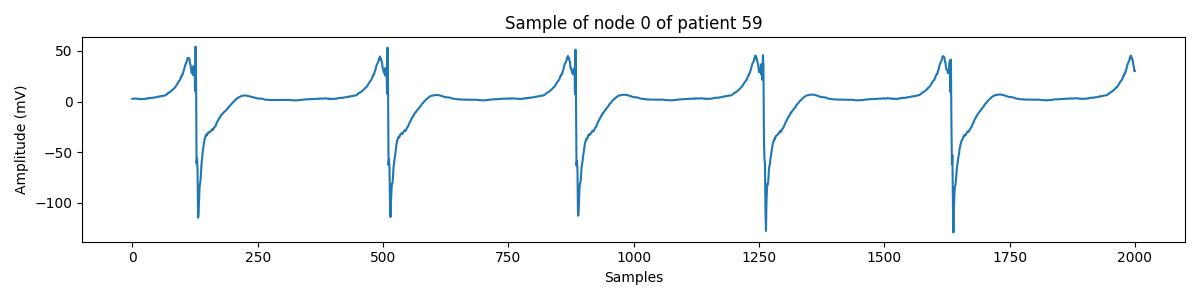

Supplement: Supplementary file 1 [file DataSheet1.zip › EGMs_sample/59.png]

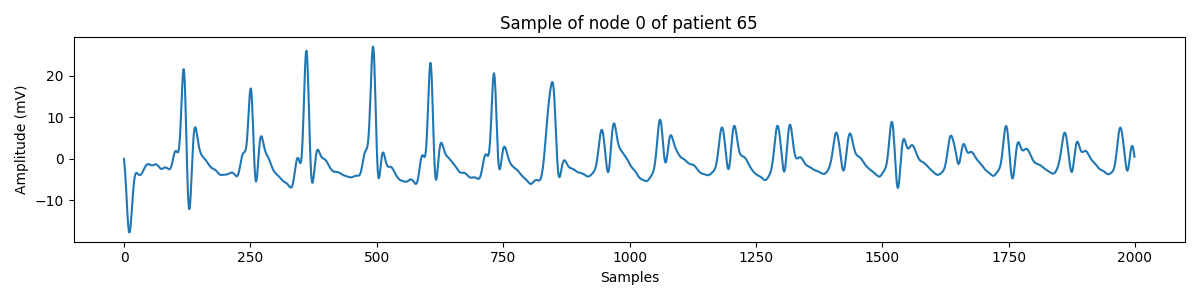

Supplement: Supplementary file 1 [file DataSheet1.zip › EGMs_sample/65.png]

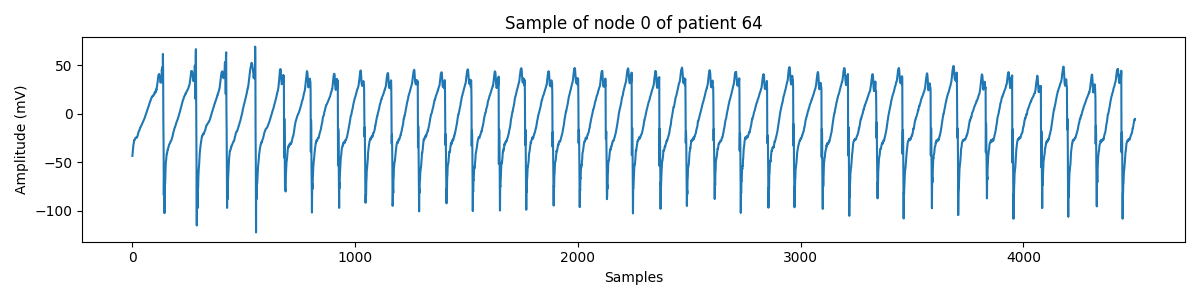

Supplement: Supplementary file 1 [file DataSheet1.zip › EGMs_sample/64.png]

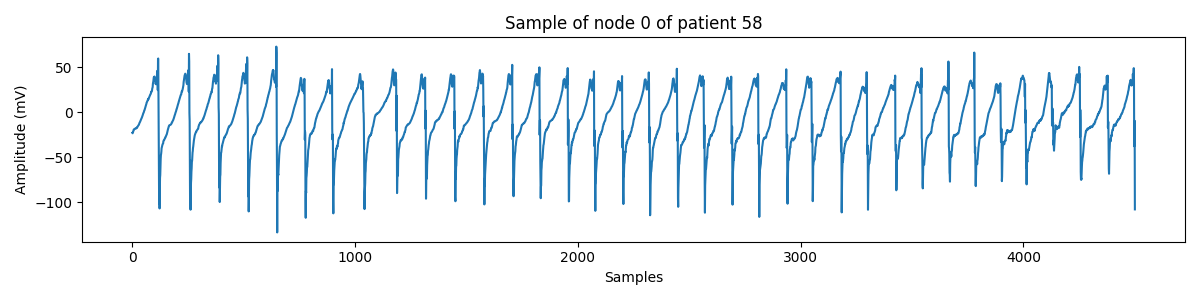

Supplement: Supplementary file 1 [file DataSheet1.zip › EGMs_sample/58.png]

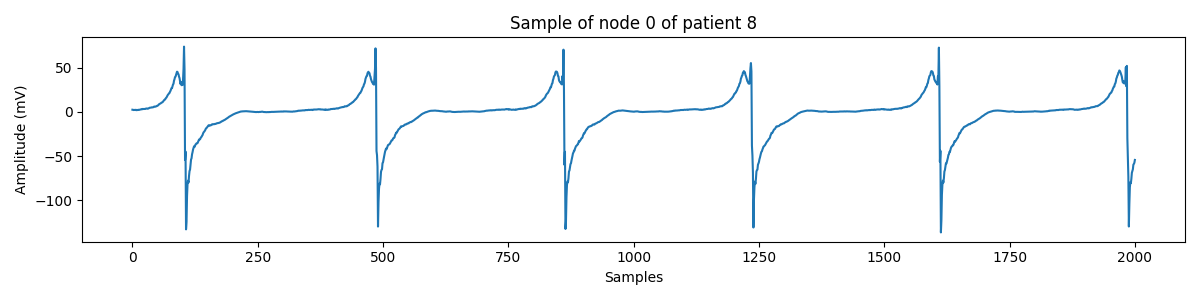

Supplement: Supplementary file 1 [file DataSheet1.zip › EGMs_sample/8.png]

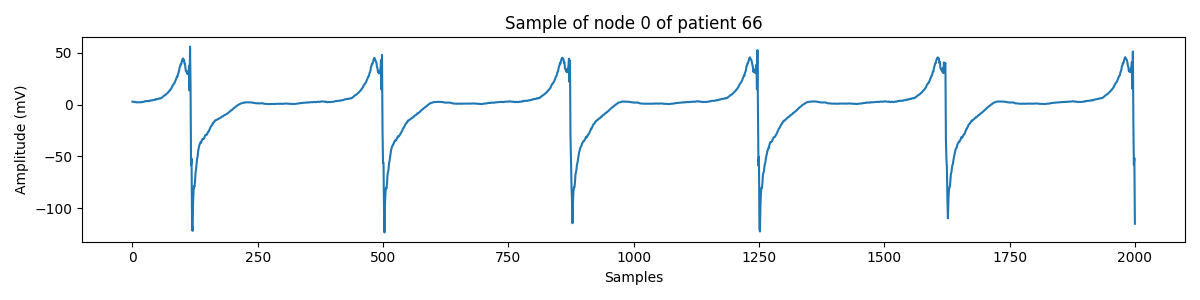

Supplement: Supplementary file 1 [file DataSheet1.zip › EGMs_sample/66.png]

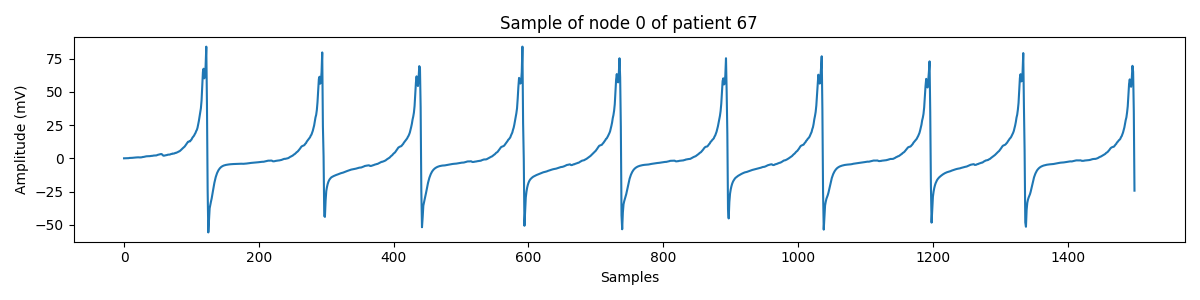

Supplement: Supplementary file 1 [file DataSheet1.zip › EGMs_sample/67.png]

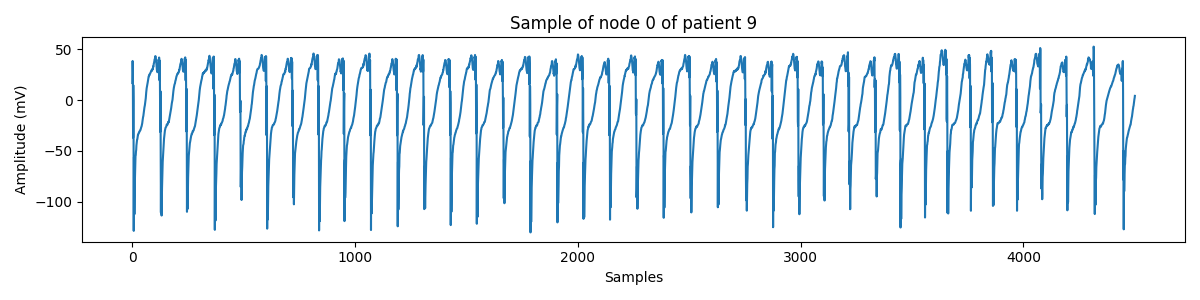

Supplement: Supplementary file 1 [file DataSheet1.zip › EGMs_sample/9.png]

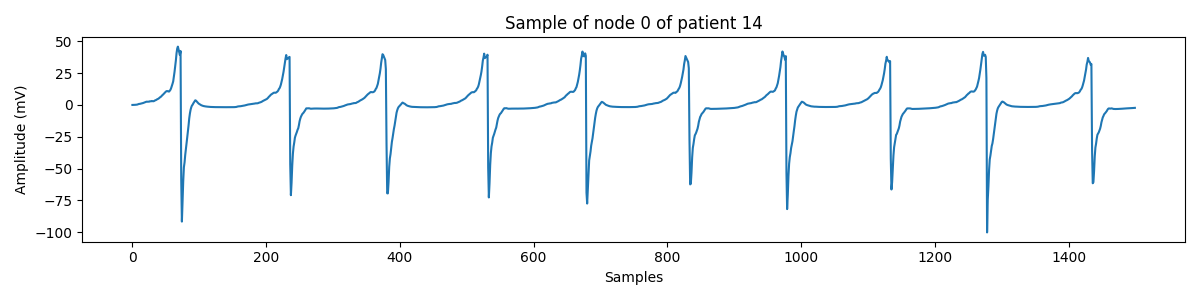

Supplement: Supplementary file 1 [file DataSheet1.zip › EGMs_sample/14.png]

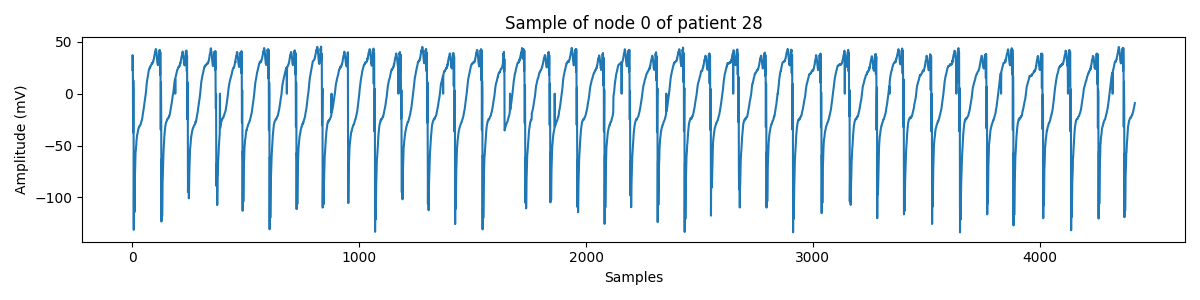

Supplement: Supplementary file 1 [file DataSheet1.zip › EGMs_sample/28.png]

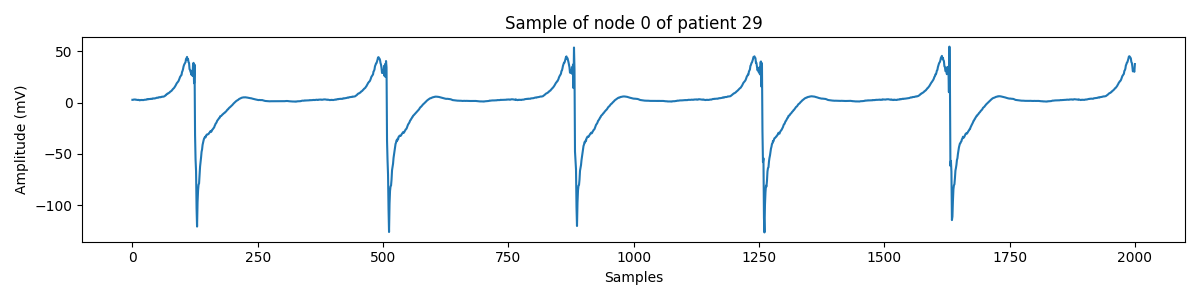

Supplement: Supplementary file 1 [file DataSheet1.zip › EGMs_sample/29.png]

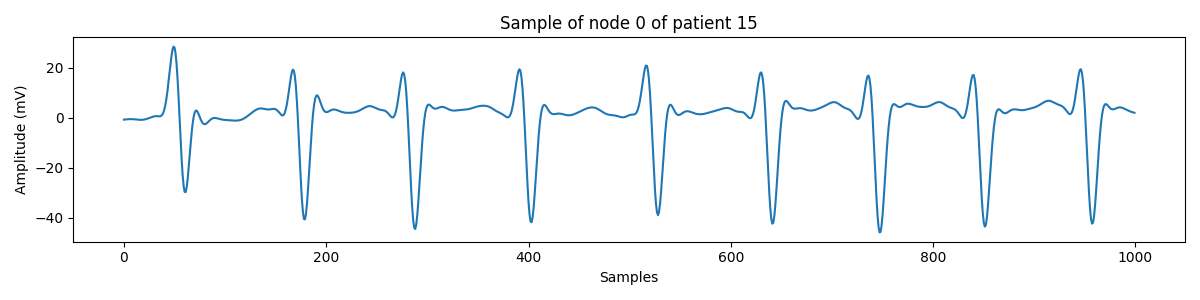

Supplement: Supplementary file 1 [file DataSheet1.zip › EGMs_sample/15.png]

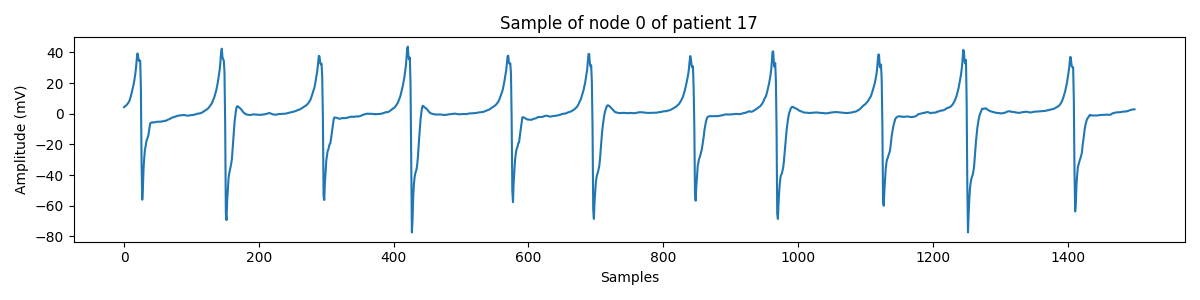

Supplement: Supplementary file 1 [file DataSheet1.zip › EGMs_sample/17.png]

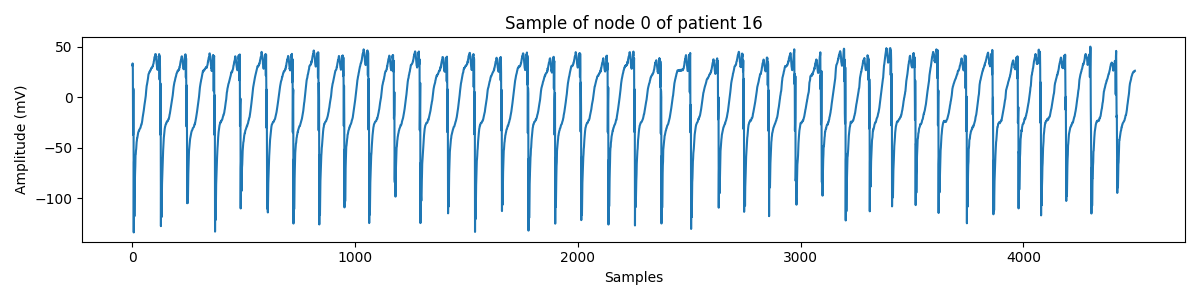

Supplement: Supplementary file 1 [file DataSheet1.zip › EGMs_sample/16.png]

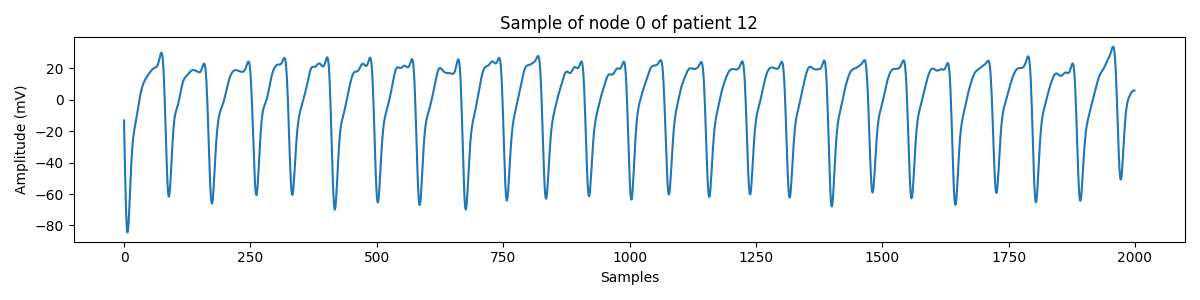

Supplement: Supplementary file 1 [file DataSheet1.zip › EGMs_sample/12.png]

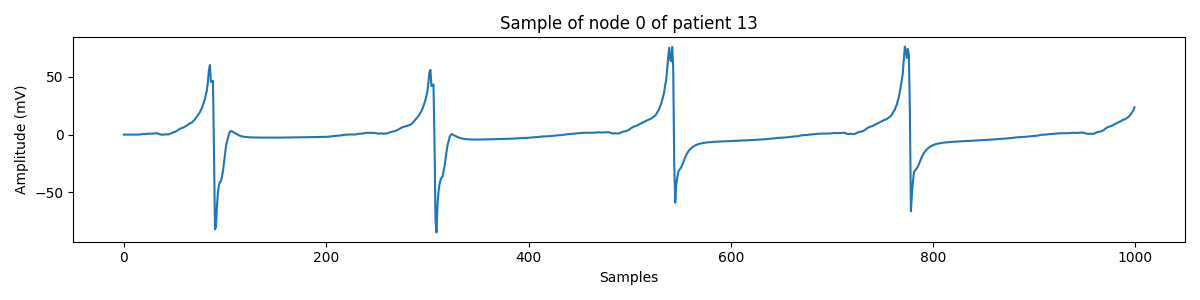

Supplement: Supplementary file 1 [file DataSheet1.zip › EGMs_sample/13.png]

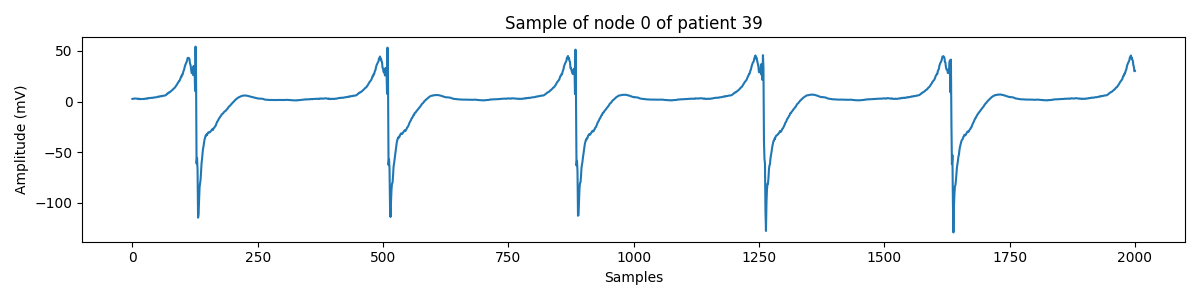

Supplement: Supplementary file 1 [file DataSheet1.zip › EGMs_sample/39.png]

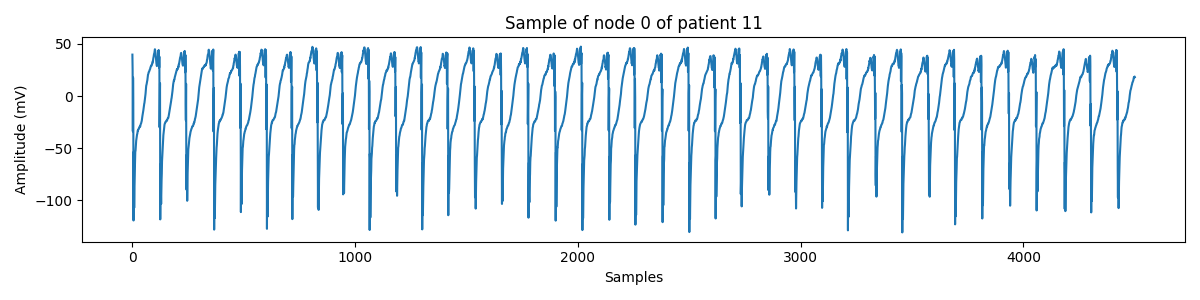

Supplement: Supplementary file 1 [file DataSheet1.zip › EGMs_sample/11.png]

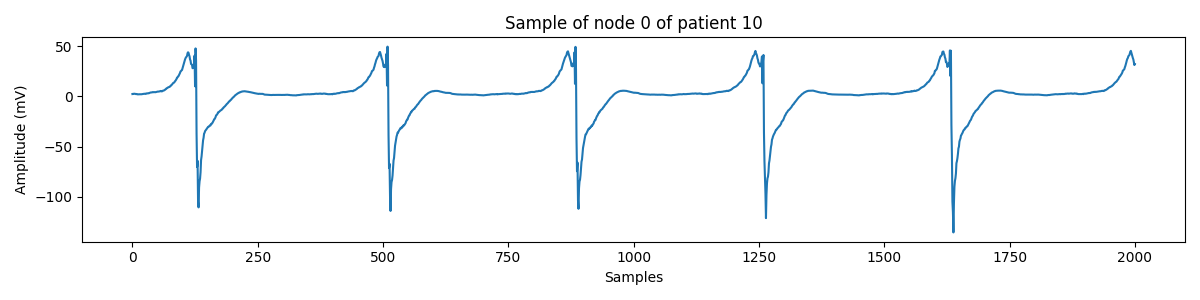

Supplement: Supplementary file 1 [file DataSheet1.zip › EGMs_sample/10.png]

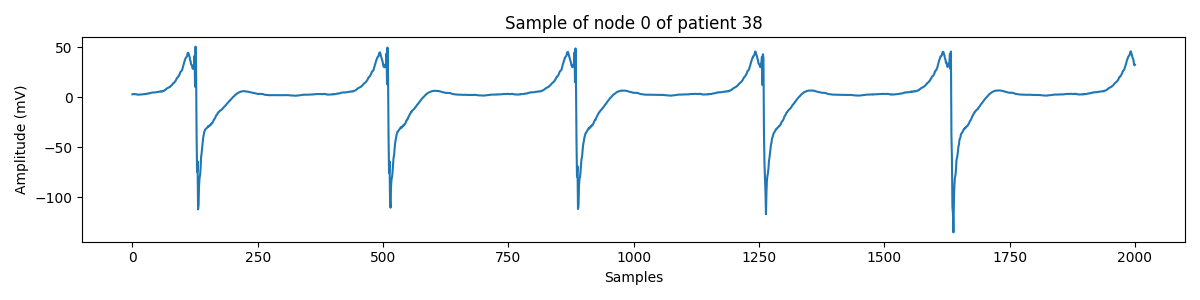

Supplement: Supplementary file 1 [file DataSheet1.zip › EGMs_sample/38.png]

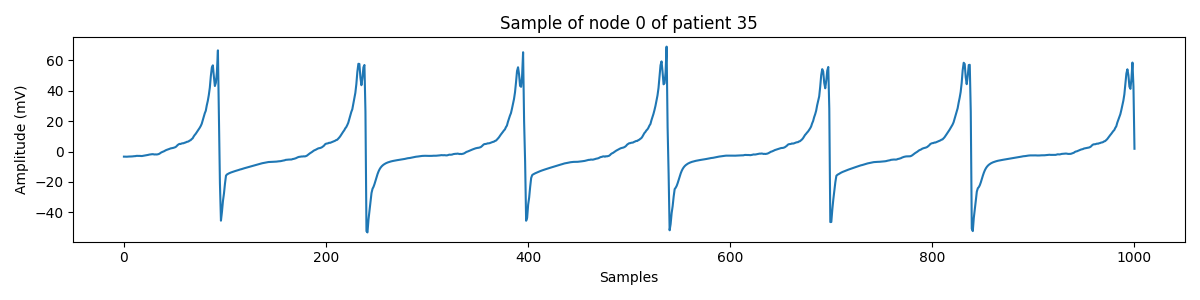

Supplement: Supplementary file 1 [file DataSheet1.zip › EGMs_sample/35.png]

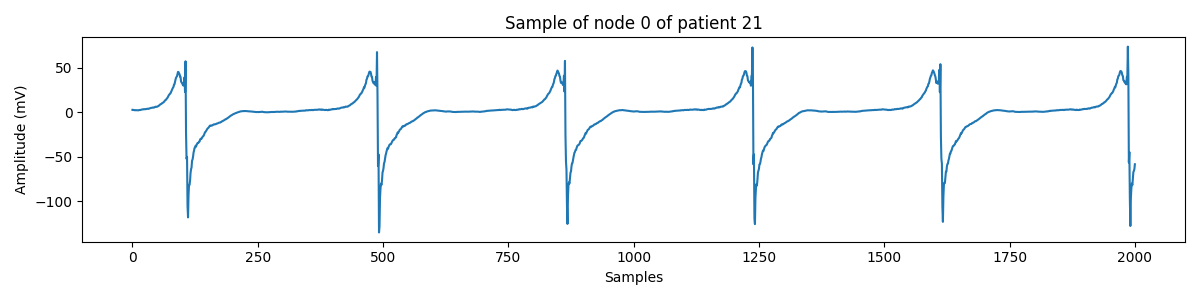

Supplement: Supplementary file 1 [file DataSheet1.zip › EGMs_sample/21.png]

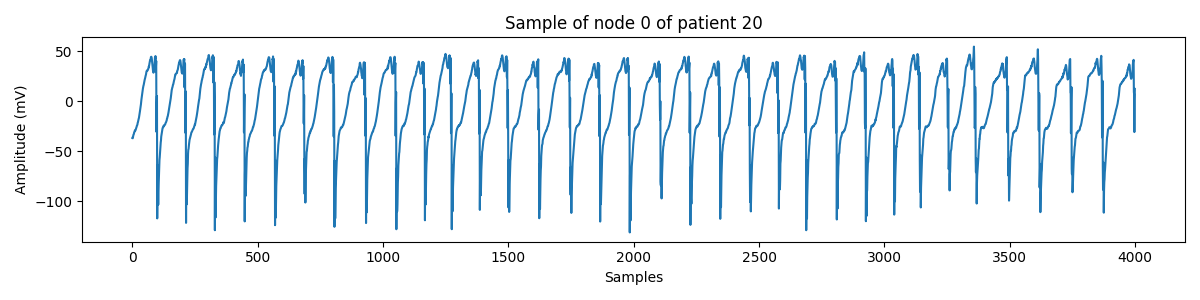

Supplement: Supplementary file 1 [file DataSheet1.zip › EGMs_sample/20.png]

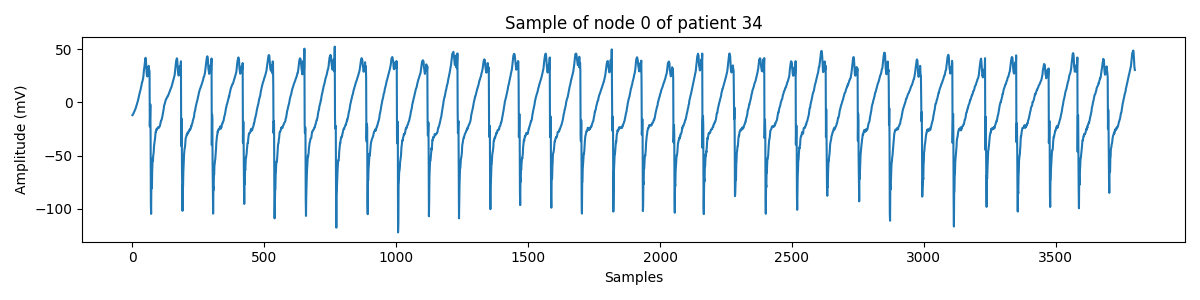

Supplement: Supplementary file 1 [file DataSheet1.zip › EGMs_sample/34.png]

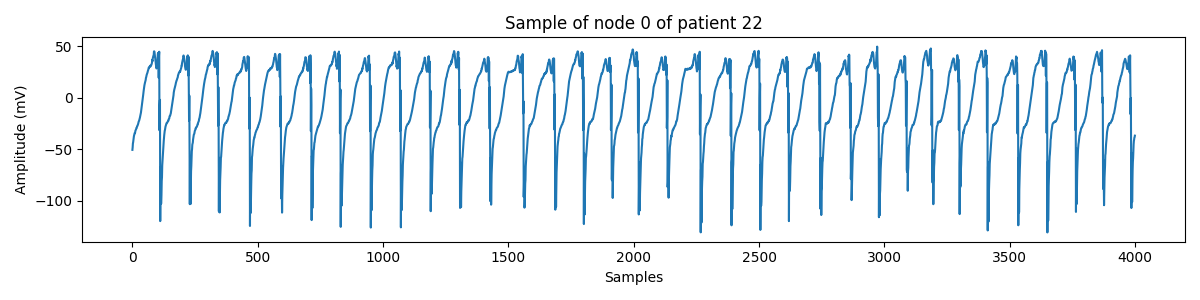

Supplement: Supplementary file 1 [file DataSheet1.zip › EGMs_sample/22.png]

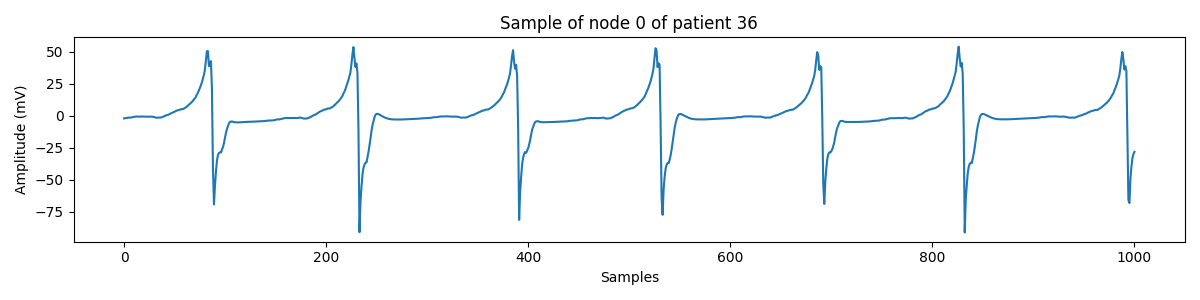

Supplement: Supplementary file 1 [file DataSheet1.zip › EGMs_sample/36.png]

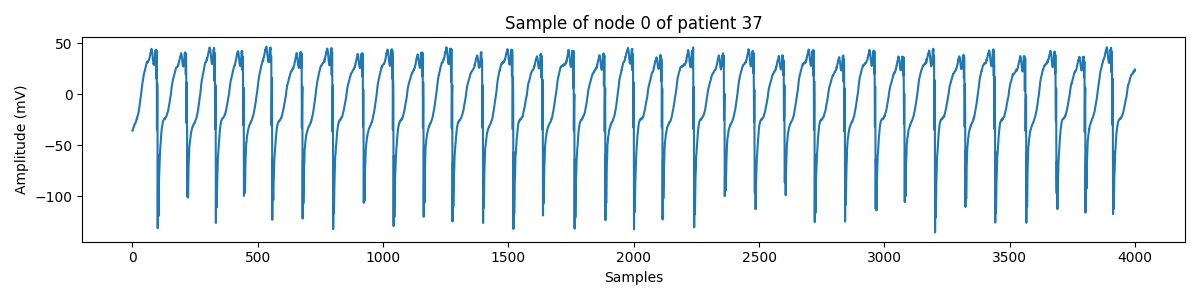

Supplement: Supplementary file 1 [file DataSheet1.zip › EGMs_sample/37.png]

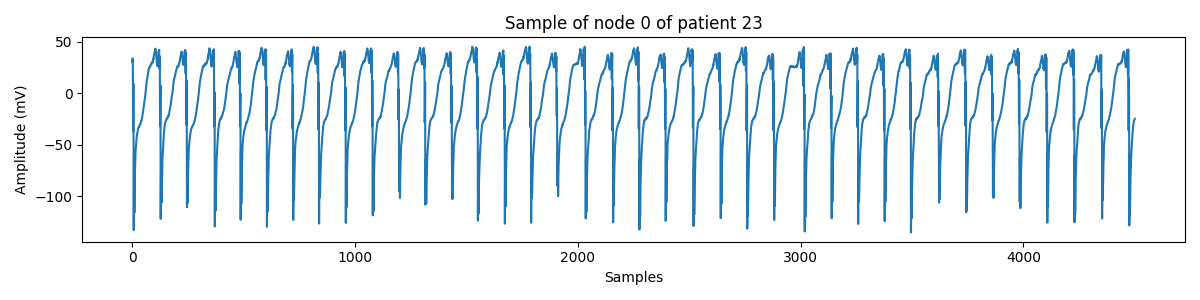

Supplement: Supplementary file 1 [file DataSheet1.zip › EGMs_sample/23.png]

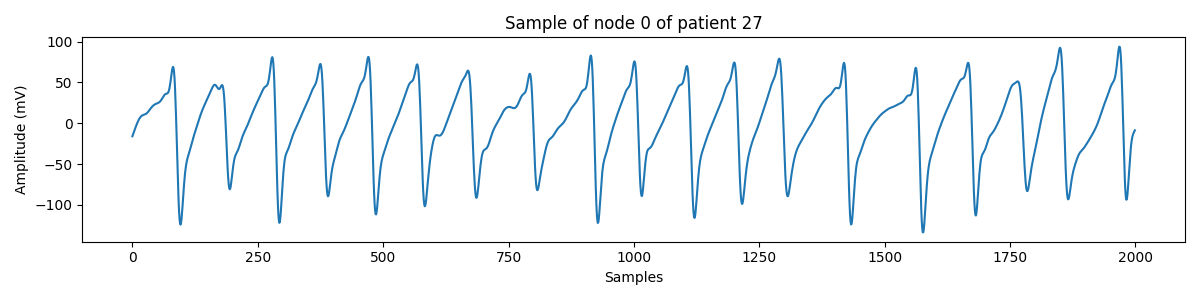

Supplement: Supplementary file 1 [file DataSheet1.zip › EGMs_sample/27.png]

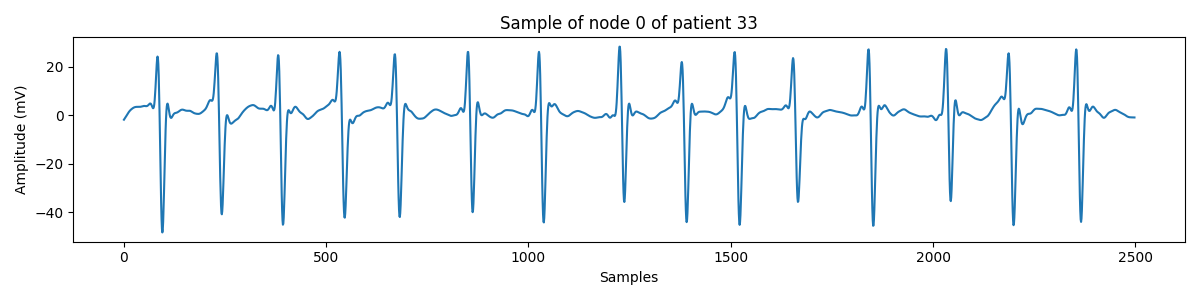

Supplement: Supplementary file 1 [file DataSheet1.zip › EGMs_sample/33.png]

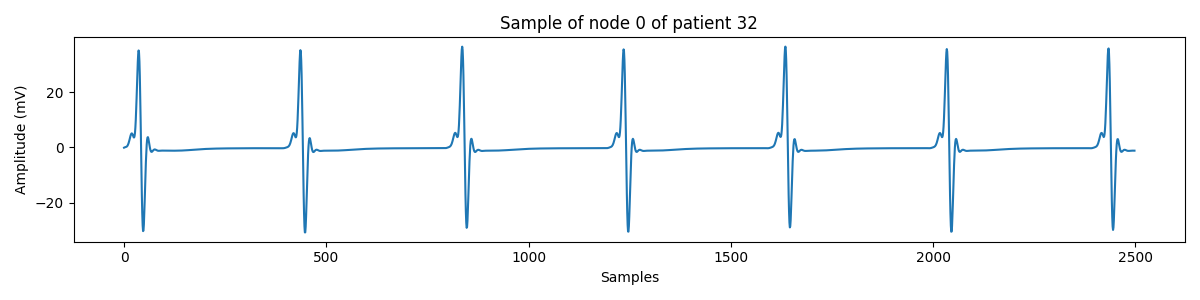

Supplement: Supplementary file 1 [file DataSheet1.zip › EGMs_sample/32.png]

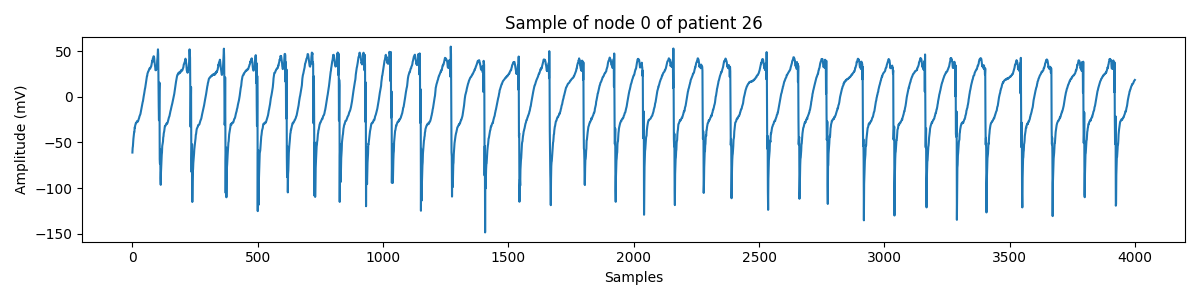

Supplement: Supplementary file 1 [file DataSheet1.zip › EGMs_sample/26.png]

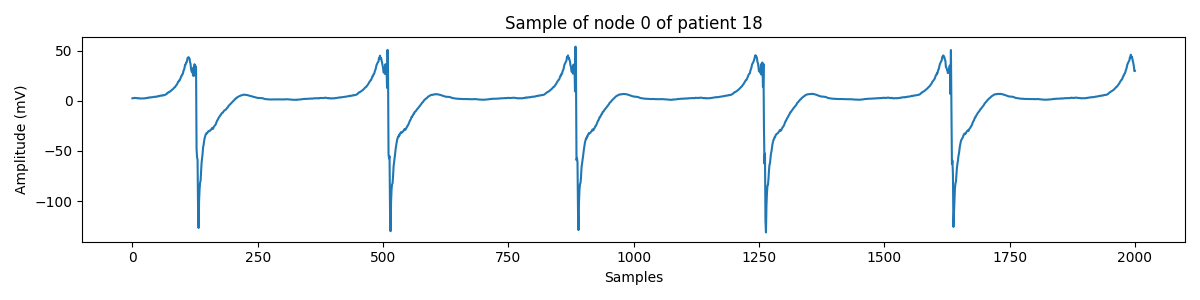

Supplement: Supplementary file 1 [file DataSheet1.zip › EGMs_sample/18.png]

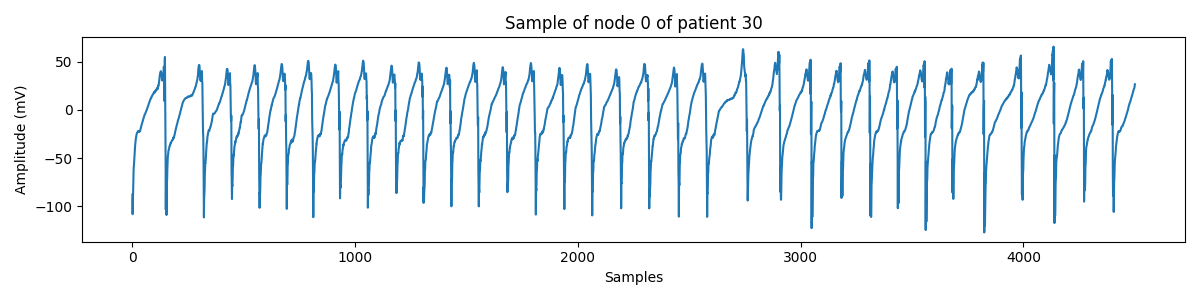

Supplement: Supplementary file 1 [file DataSheet1.zip › EGMs_sample/30.png]

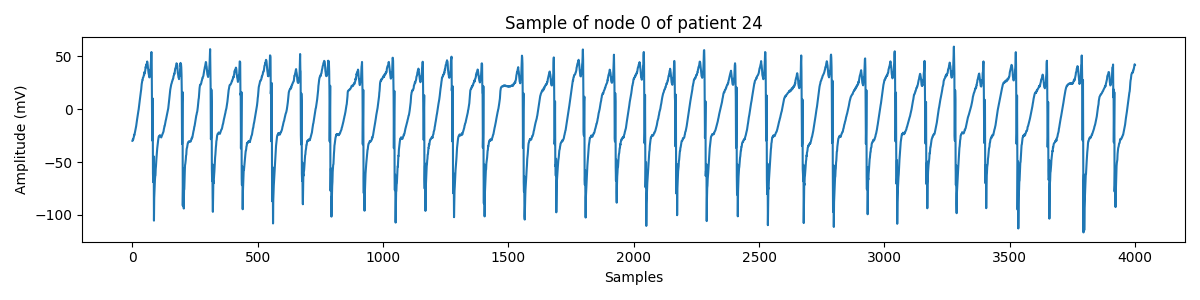

Supplement: Supplementary file 1 [file DataSheet1.zip › EGMs_sample/24.png]

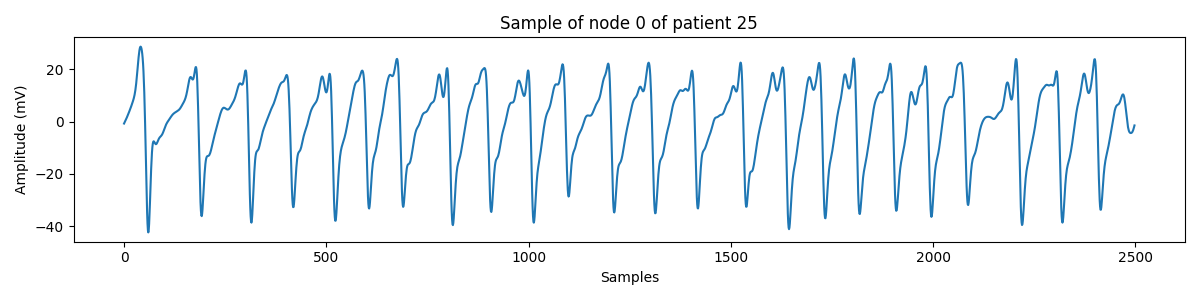

Supplement: Supplementary file 1 [file DataSheet1.zip › EGMs_sample/25.png]

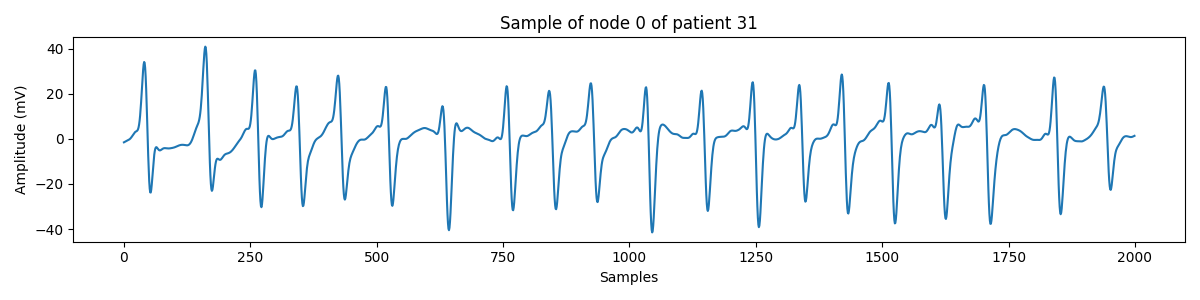

Supplement: Supplementary file 1 [file DataSheet1.zip › EGMs_sample/31.png]

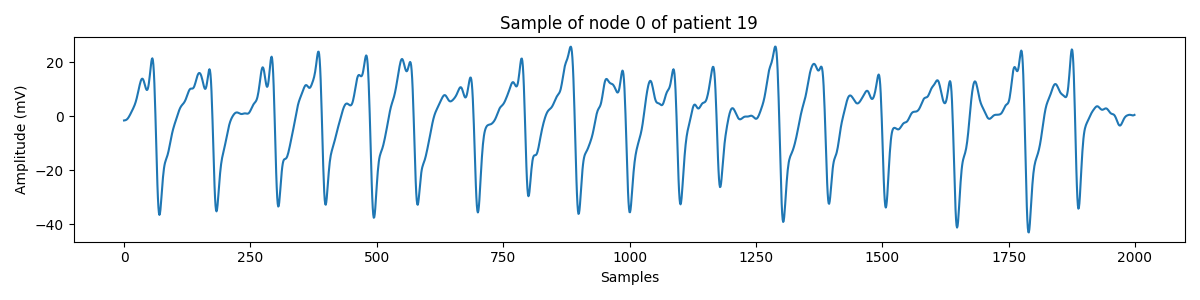

Supplement: Supplementary file 1 [file DataSheet1.zip › EGMs_sample/19.png]

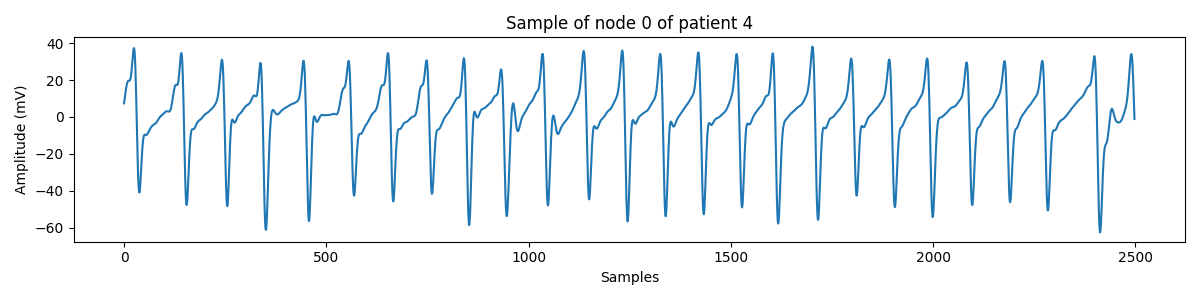

Supplement: Supplementary file 1 [file DataSheet1.zip › EGMs_sample/4.png]

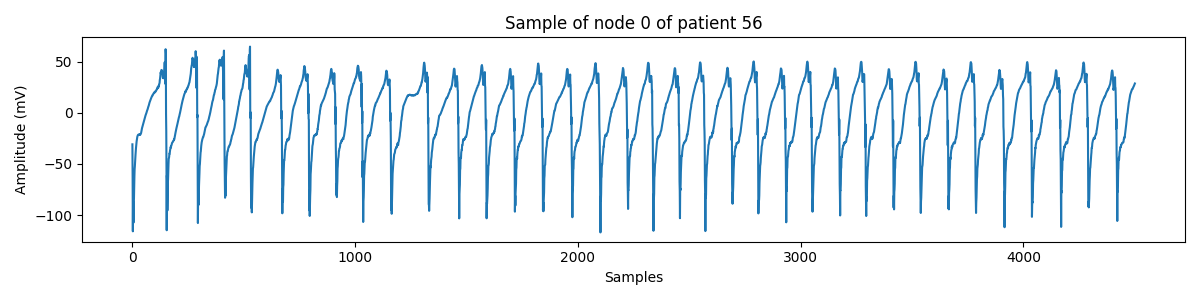

Supplement: Supplementary file 1 [file DataSheet1.zip › EGMs_sample/56.png]

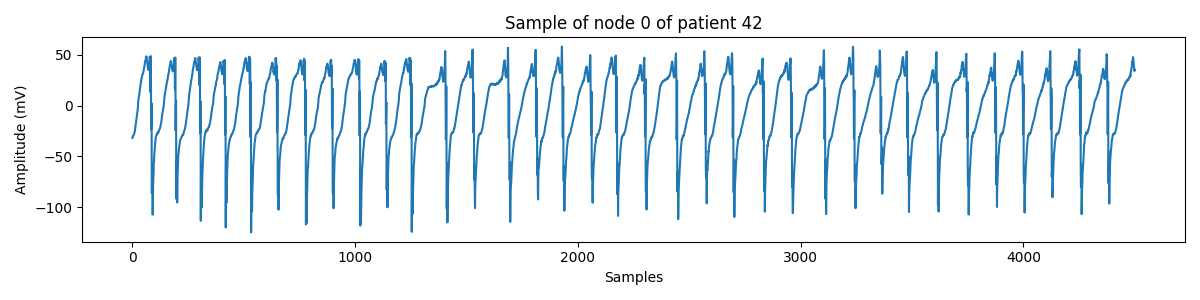

Supplement: Supplementary file 1 [file DataSheet1.zip › EGMs_sample/42.png]

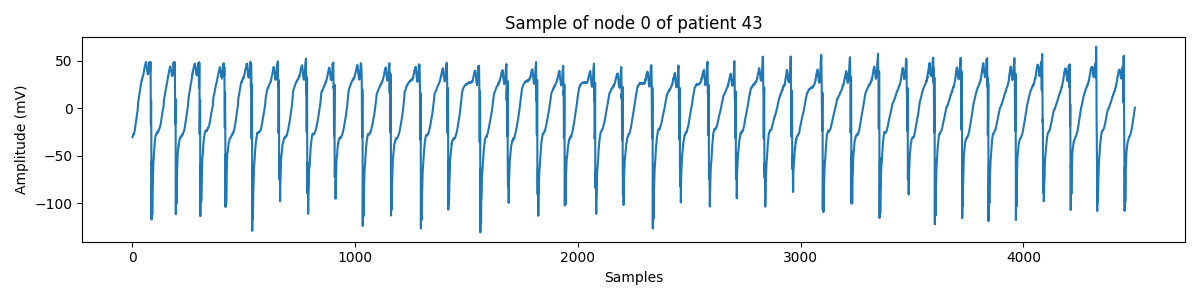

Supplement: Supplementary file 1 [file DataSheet1.zip › EGMs_sample/43.png]

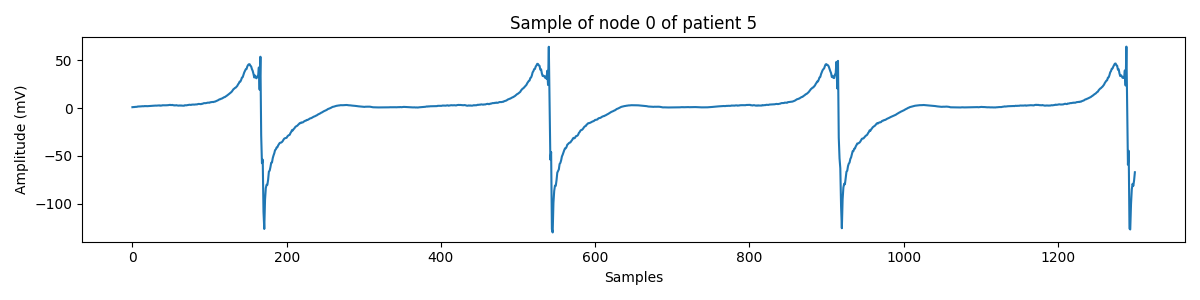

Supplement: Supplementary file 1 [file DataSheet1.zip › EGMs_sample/5.png]

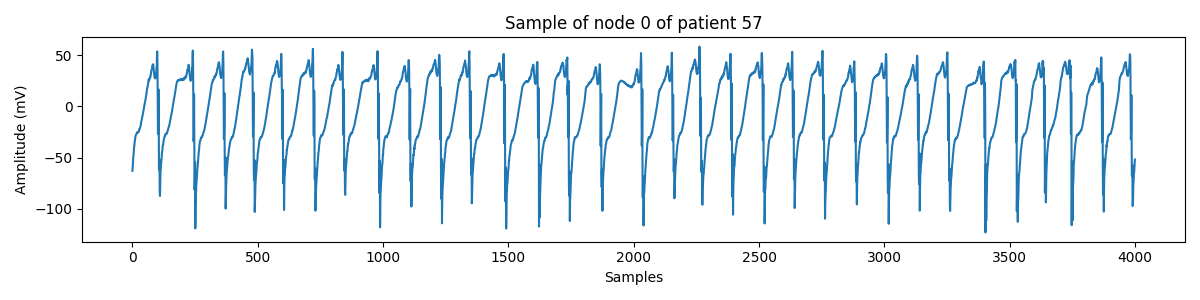

Supplement: Supplementary file 1 [file DataSheet1.zip › EGMs_sample/57.png]

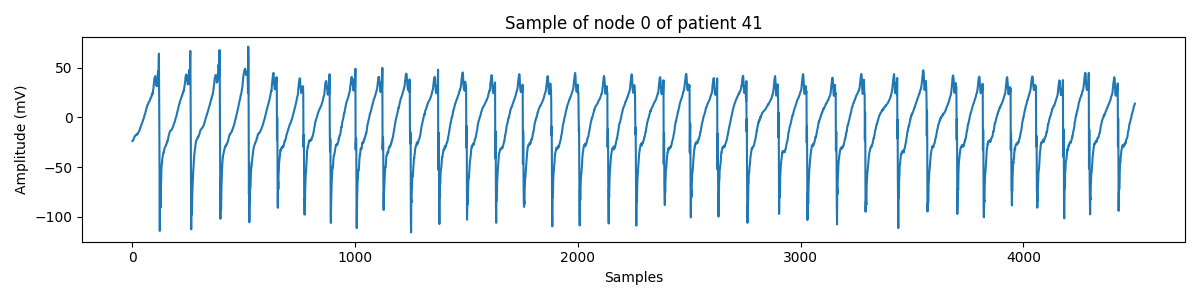

Supplement: Supplementary file 1 [file DataSheet1.zip › EGMs_sample/41.png]

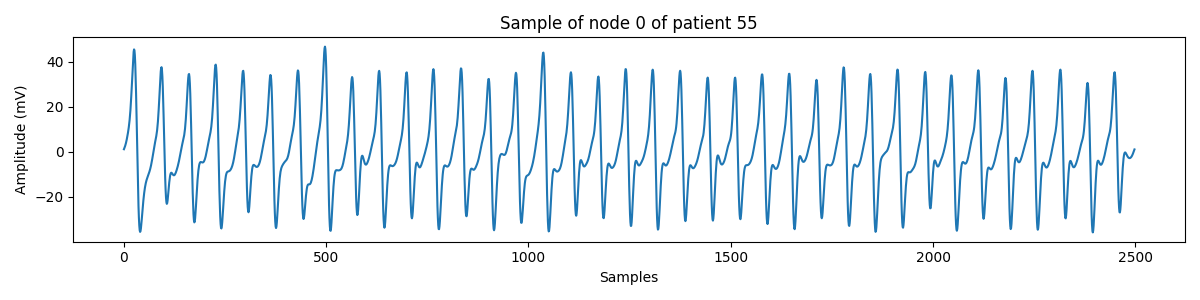

Supplement: Supplementary file 1 [file DataSheet1.zip › EGMs_sample/55.png]

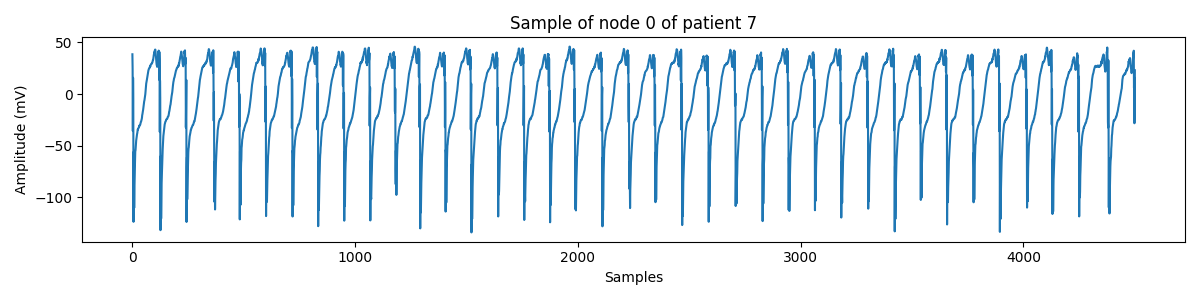

Supplement: Supplementary file 1 [file DataSheet1.zip › EGMs_sample/7.png]

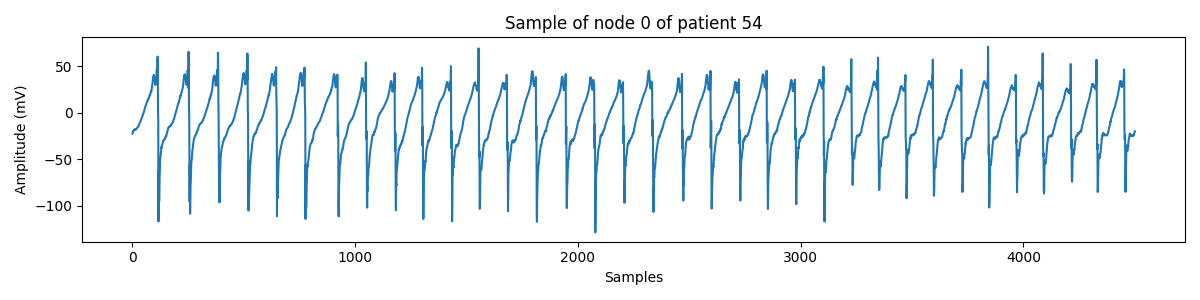

Supplement: Supplementary file 1 [file DataSheet1.zip › EGMs_sample/54.png]

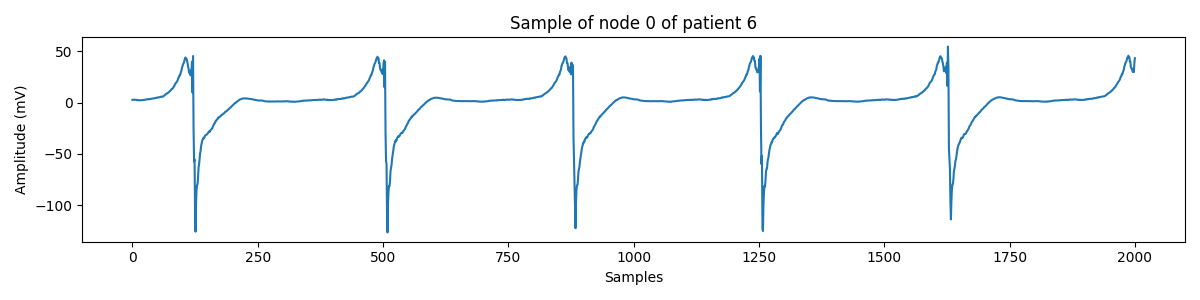

Supplement: Supplementary file 1 [file DataSheet1.zip › EGMs_sample/6.png]

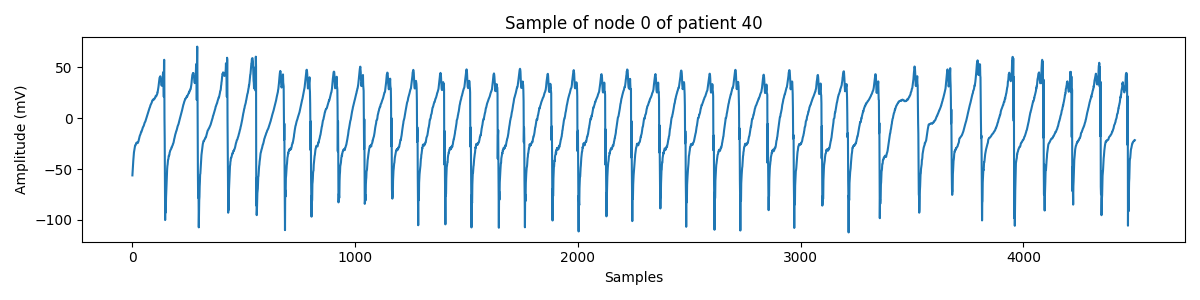

Supplement: Supplementary file 1 [file DataSheet1.zip › EGMs_sample/40.png]

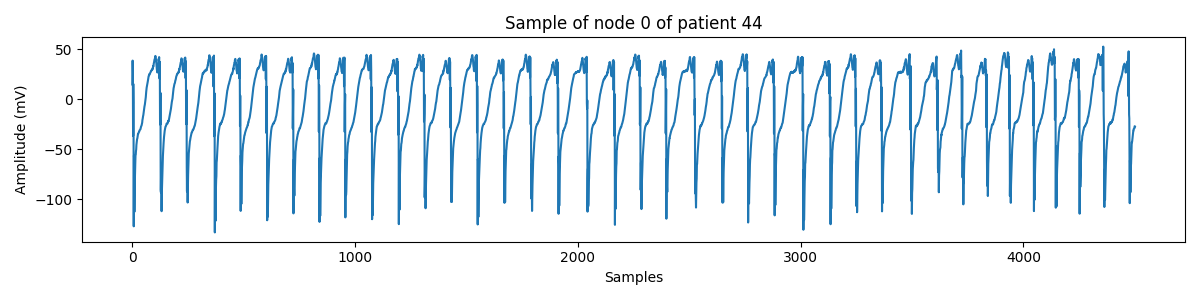

Supplement: Supplementary file 1 [file DataSheet1.zip › EGMs_sample/44.png]

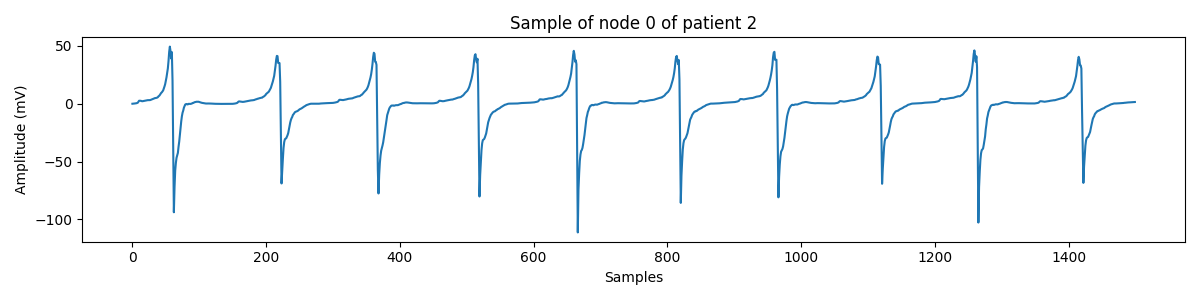

Supplement: Supplementary file 1 [file DataSheet1.zip › EGMs_sample/2.png]

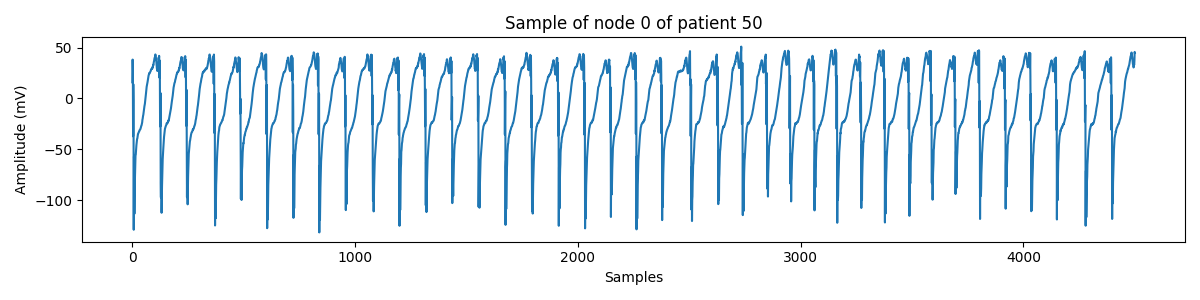

Supplement: Supplementary file 1 [file DataSheet1.zip › EGMs_sample/50.png]

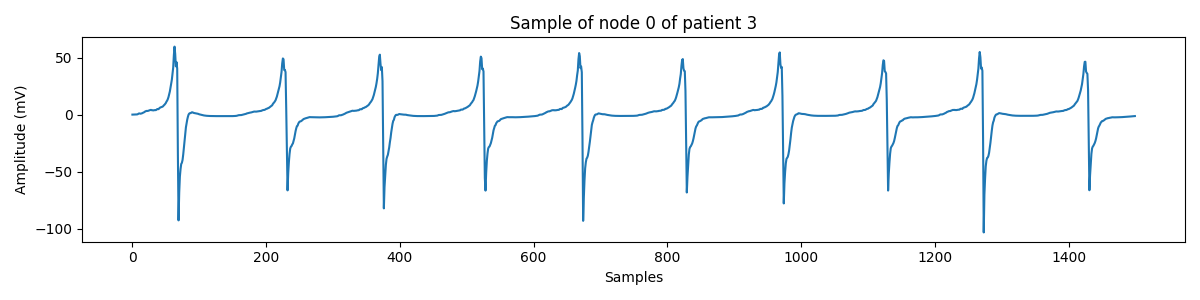

Supplement: Supplementary file 1 [file DataSheet1.zip › EGMs_sample/3.png]

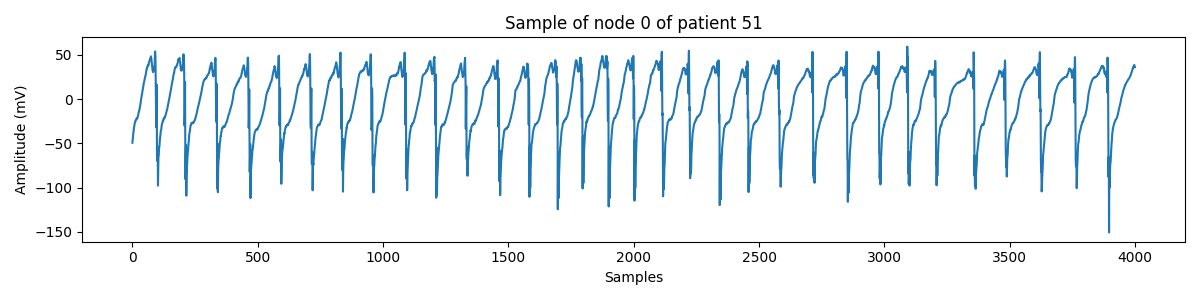

Supplement: Supplementary file 1 [file DataSheet1.zip › EGMs_sample/51.png]

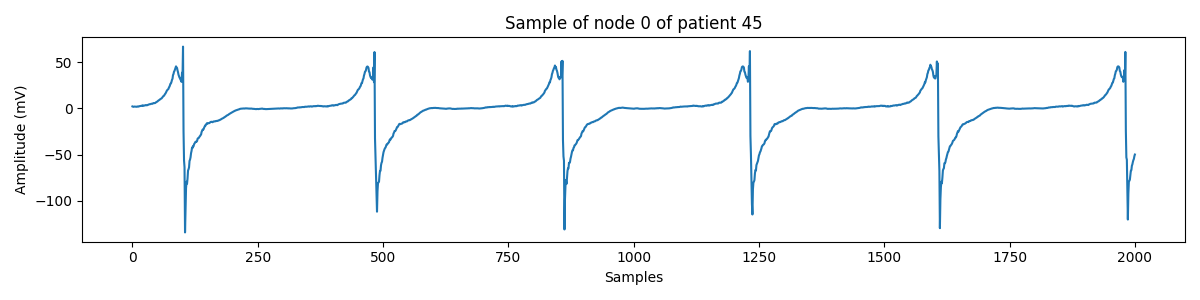

Supplement: Supplementary file 1 [file DataSheet1.zip › EGMs_sample/45.png]

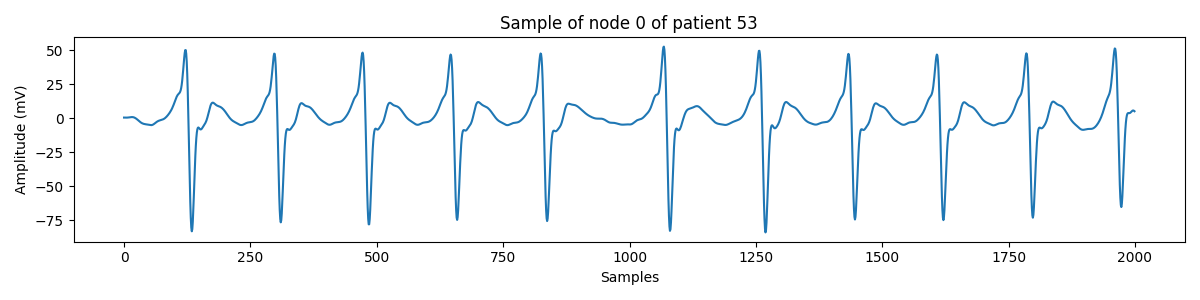

Supplement: Supplementary file 1 [file DataSheet1.zip › EGMs_sample/53.png]

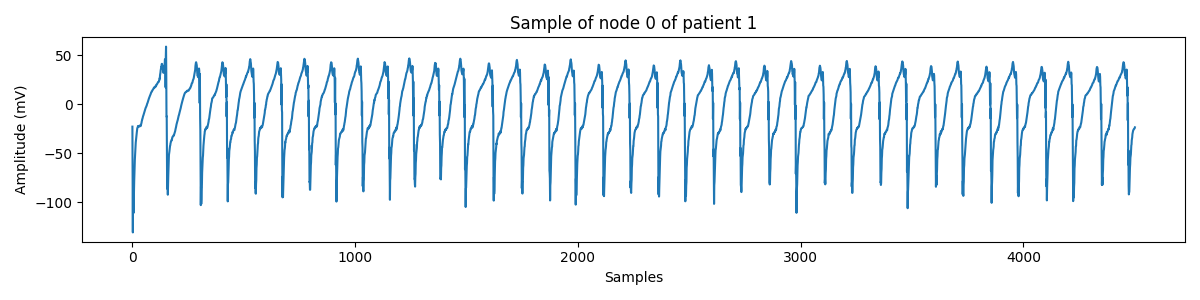

Supplement: Supplementary file 1 [file DataSheet1.zip › EGMs_sample/1.png]

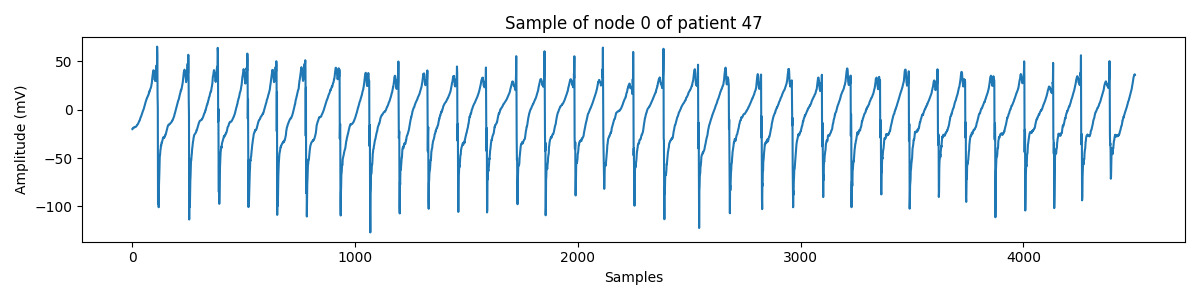

Supplement: Supplementary file 1 [file DataSheet1.zip › EGMs_sample/47.png]

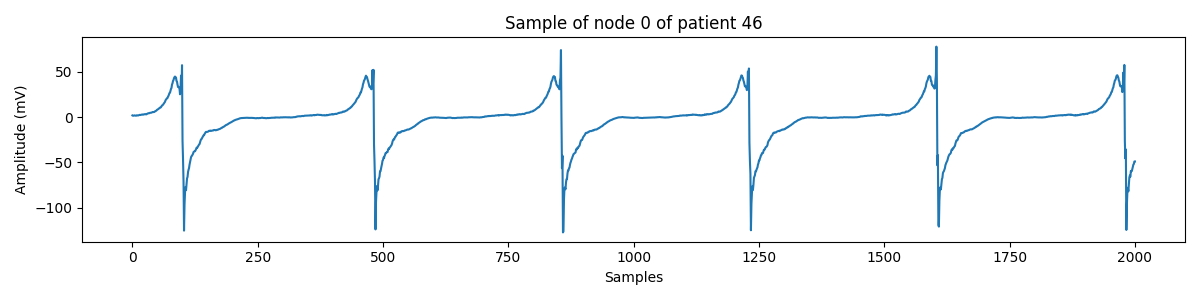

Supplement: Supplementary file 1 [file DataSheet1.zip › EGMs_sample/46.png]

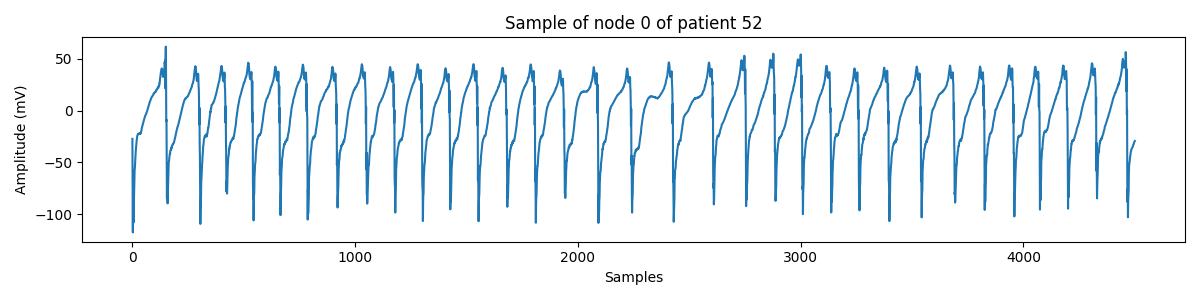

Supplement: Supplementary file 1 [file DataSheet1.zip › EGMs_sample/52.png]

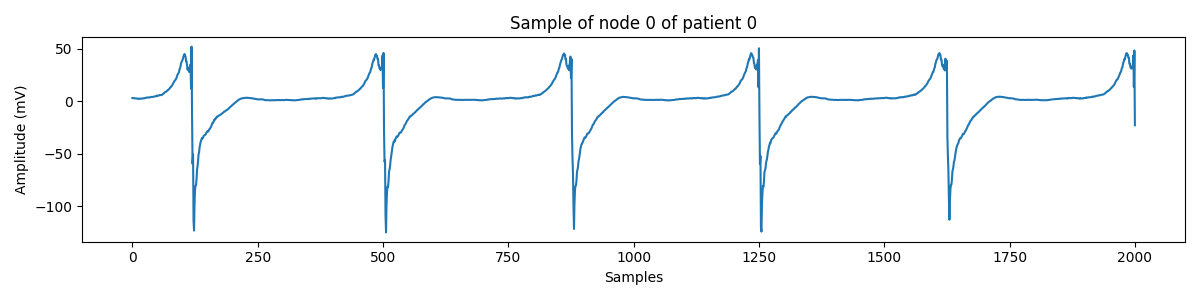

Supplement: Supplementary file 1 [file DataSheet1.zip › EGMs_sample/0.png]
