# Supplementary material for: Deep learning for atrial electrogram estimation: toward non-invasive arrhythmia mapping using variational autoencoders
Source: Front Physiol. 2026 Jan 12;16:1720244. doi: 10.3389/fphys.2025.1720244 (PMC12832759; doi:10.3389/fphys.2025.1720244)
Supplement: Supplementary file 2 [file Supplementaryfile1.pdf]

Supplementary Material

1 SUPPLEMENTARY TABLES AND FIGURES

1.1 Tables

Table S1. Results of ZOT for all 12 test patients

id patient	Correlation	RMSE	Peak detector Precision	DTW	Coherence
LA_PLAW_140711_arm	0.09	0.55	0.78	112.18	0.68
LA_RSPV_CAF_150115	0.06	0.59	0.59	110.69	0.45
Simulation_01_200212_001_5	0.01	0.66	0.76	101.45	0.86
Simulation_01_200212_001_10	0.05	0.58	0.63	226.04	0.27
Simulation_01_200316_001_3	0.03	0.66	0.78	102.44	0.85
Simulation_01_200316_001_4	0.08	0.56	0.63	212.96	0.29
Simulation_01_200316_001_8	0.09	0.58	0.65	218.81	0.34
Simulation_01_200428_001_004	0.09	0.54	0.65	211.09	0.31
Simulation_01_200428_001_008	0.09	0.55	0.65	209.82	0.29
Simulation_01_200428_001_010	0.09	0.54	0.65	203.58	0.26
Simulation_01_210119_001_001	0.40	0.55	0.84	56.15	1.00
Simulation_01_210208_001_002	0.46	0.58	0.74	61.95	1.00

Table S2. Results of DL best-performing model for all 12 test patients

patient id	Correlation	RMSE	Peak detector Precision	DTW	Coherence
LA_PLAW_140711_arm	-0.00	0.60	0.70	118.61	0.40
LA_RSPV_CAF_150115	0.01	0.61	0.48	118.07	0.37
Simulation_01_200212_001_5	0.56	0.51	0.96	106.89	0.85
Simulation_01_200212_001_10	0.41	0.53	0.70	220.57	0.24
Simulation_01_200316_001_3	0.57	0.51	0.96	105.78	0.84
Simulation_01_200316_001_4	0.46	0.59	0.69	215.83	0.29
Simulation_01_200316_001_8	0.27	0.61	0.66	231.92	0.21
Simulation_01_200428_001_004	0.51	0.57	0.74	211.25	0.31
Simulation_01_200428_001_008	0.55	0.56	0.81	218.65	0.34
Simulation_01_200428_001_010	0.37	0.57	0.66	214.78	0.26
Simulation_01_210119_001_001	0.01	0.60	0.89	66.56	1.00
Simulation_01_210208_001_002	0.01	0.60	0.69	58.08	1.00

Table S3. Combined statistical summary including DL and ZOT descriptive statistics and paired bootstrap comparison.

Metric	DL (mean±sd)	ZOT (mean±sd)	Δ(mean)	95% CI	DL better than ZOT
Correlation	0.310±0.240	0.130±0.145	0.1825	[0.0175, 0.3342]	Yes
RMSE	0.573±0.037	0.565±0.042	-0.0067	[-0.0417, 0.0242]	No
Peak Precision	0.745±0.142	0.736±0.083	0.0492	[0.0033, 0.0950]	Yes
DTW	157.250±66.937	244.587±134.403	4.9858	[2.2642, 7.5800]	No
Coherence	0.509±0.312	0.536±0.315	-0.0408	[-0.0842, -0.0050]	No

Table S4. Ionic and tissue parameters used in the atrial simulations. Ionic currents follow the Nygren et al. (1998) model with Koivumäki et al. (2014) Ca^{2+} handling.

Parameter	Description	Value
**Membrane & Ionic Model Parameters**		
C_m	Membrane capacitance	$1.0\ \mu\mathrm{F/cm}^2$
g_{Na}	Fast Na^+ current conductance	$7.8\ \mathrm{nS/pF}$
g_{CaL}	L-type Ca^{2+} current conductance	$0.1238\ \mathrm{nS/pF}$
g_{to}	Transient outward K^+ current	$0.1652\ \mathrm{nS/pF}$
g_{Ks}	Slow delayed rectifier K^+ current	$0.05\ \mathrm{nS/pF}$
g_{Kr}	Rapid delayed rectifier K^+ current	$0.0294\ \mathrm{nS/pF}$
g_{K1}	Inward rectifier K^+ current	$0.09\ \mathrm{nS/pF}$
g_{NaK}	Na^+/K^+ pump	$0.06441\ \mathrm{pA/pF}$
g_{NCX}	Na^+/Ca^{2+} exchanger	$2.5\ \mathrm{pA/pF}$
V_{leak}	SR Ca^{2+} leak (Koivumäki)	$0.005\ \mathrm{ms}^{-1}$
V_{rel}	SR Ca^{2+} release (Koivumäki)	$0.1\ \mathrm{ms}^{-1}$
V_{uptake}	SERCA uptake rate (Koivumäki)	$0.006\ \mathrm{ms}^{-1}$
**Tissue-Level Parameters**		
D_{long}	Longitudinal diffusion coefficient	$0.20\ \mathrm{mm^2/ms}$
D_{trans}	Transverse diffusion coefficient	$0.05\ \mathrm{mm^2/ms}$
CV ratio	Anisotropy ratio (long:trans)	4:1
Fibrosis model	Nodes with zero diffusion	$D = 0$
Δt	Time step	$0.02\ \mathrm{ms}$
Mesh neighbors	Coupling stencil	1-ring adjacency

1.2 Figures

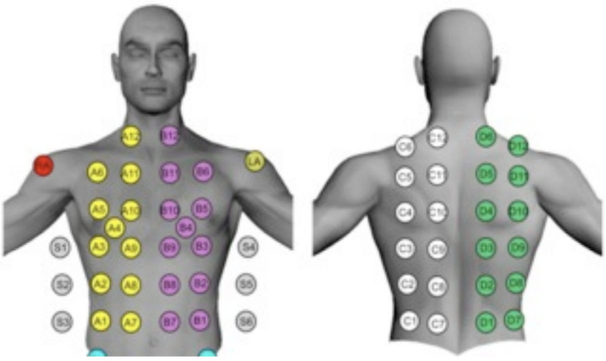

Figure S1. Electrode position covering the torso used for reference. Taken from Pedrón-Torrecilla et al. (2016)

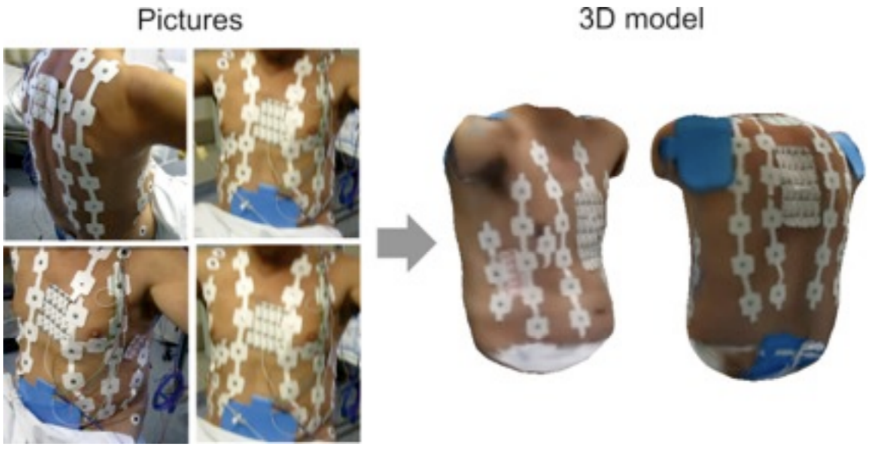

Figure S2. Surface electrical signals recorded in 54 leads covering the torso used for reference. Taken from Pedrón-Torrecilla et al. (2016)

Layer (type)	Output Shape	Param #	Connected to
input_1 (InputLayer)	[(None, 400, 12, 32, 1)]	0	[]
conv3d (Conv3D)	(None, 400, 12, 32, 64)	1344	['input_1[0][0]']
conv3d_1 (Conv3D)	(None, 400, 12, 32, 64)	81984	['conv3d[0][0]']
conv3d_2 (Conv3D)	(None, 400, 12, 32, 32)	40992	['conv3d_1[0][0]']
max_pooling3d (MaxPooling3D)	(None, 400, 6, 16, 32)	0	['conv3d_2[0][0]']
conv3d_3 (Conv3D)	(None, 400, 6, 16, 12)	7692	['max_pooling3d[0][0]']
max_pooling3d_1 (MaxPooling3D)	(None, 400, 3, 8, 12)	0	['conv3d_3[0][0]']
conv3d_4 (Conv3D)	(None, 400, 3, 8, 12)	2892	['max_pooling3d_1[0][0]']
max_pooling3d_2 (MaxPooling3D)	(None, 400, 3, 4, 12)	0	['conv3d_4[0][0]']
conv3d_8 (Conv3D)	(None, 400, 3, 4, 64)	15424	['max_pooling3d_2[0][0]']
up_sampling3d_3 (UpSampling3D)	(None, 400, 6, 8, 64)	0	['conv3d_8[0][0]']
conv3d_9 (Conv3D)	(None, 400, 6, 8, 32)	92192	['up_sampling3d_3[0][0]']
conv3d_5 (Conv3D)	(None, 400, 3, 4, 12)	2892	['max_pooling3d_2[0][0]']
up_sampling3d_4 (UpSampling3D)	(None, 400, 12, 16, 32)	0	['conv3d_9[0][0]']
up_sampling3d (UpSampling3D)	(None, 400, 3, 8, 12)	0	['conv3d_5[0][0]']
conv3d_10 (Conv3D)	(None, 400, 12, 16, 3)	4323	['up_sampling3d_4[0][0]']
conv3d_6 (Conv3D)	(None, 400, 3, 8, 32)	7712	['up_sampling3d[0][0]']
time_distributed (TimeDistributed)	(None, 400, 576)	0	['conv3d_10[0][0]']
up_sampling3d_1 (UpSampling3D)	(None, 400, 6, 16, 32)	0	['conv3d_6[0][0]']
batch_normalization (BatchNormalization)	(None, 400, 576)	1600	['time_distributed[0][0]']
conv3d_7 (Conv3D)	(None, 400, 6, 16, 32)	20512	['up_sampling3d_1[0][0]']
lstm (LSTM)	(None, 400, 40)	98720	['batch_normalization[0][0]']
up_sampling3d_2 (UpSampling3D)	(None, 400, 12, 32, 32)	0	['conv3d_7[0][0]']
dropout (Dropout)	(None, 400, 40)	0	['lstm[0][0]']
autoencoder (Conv3D)	(None, 400, 12, 32, 1)	641	['up_sampling3d_2[0][0]']
reconstruction (Dense)	(None, 400, 2048)	83968	['dropout[0][0]']

Total params: 462888 (1.77 MB)
Trainable params: 462088 (1.76 MB)
Non-trainable params: 800 (3.12 KB)

Figure S3. Model summary of layers and parameters

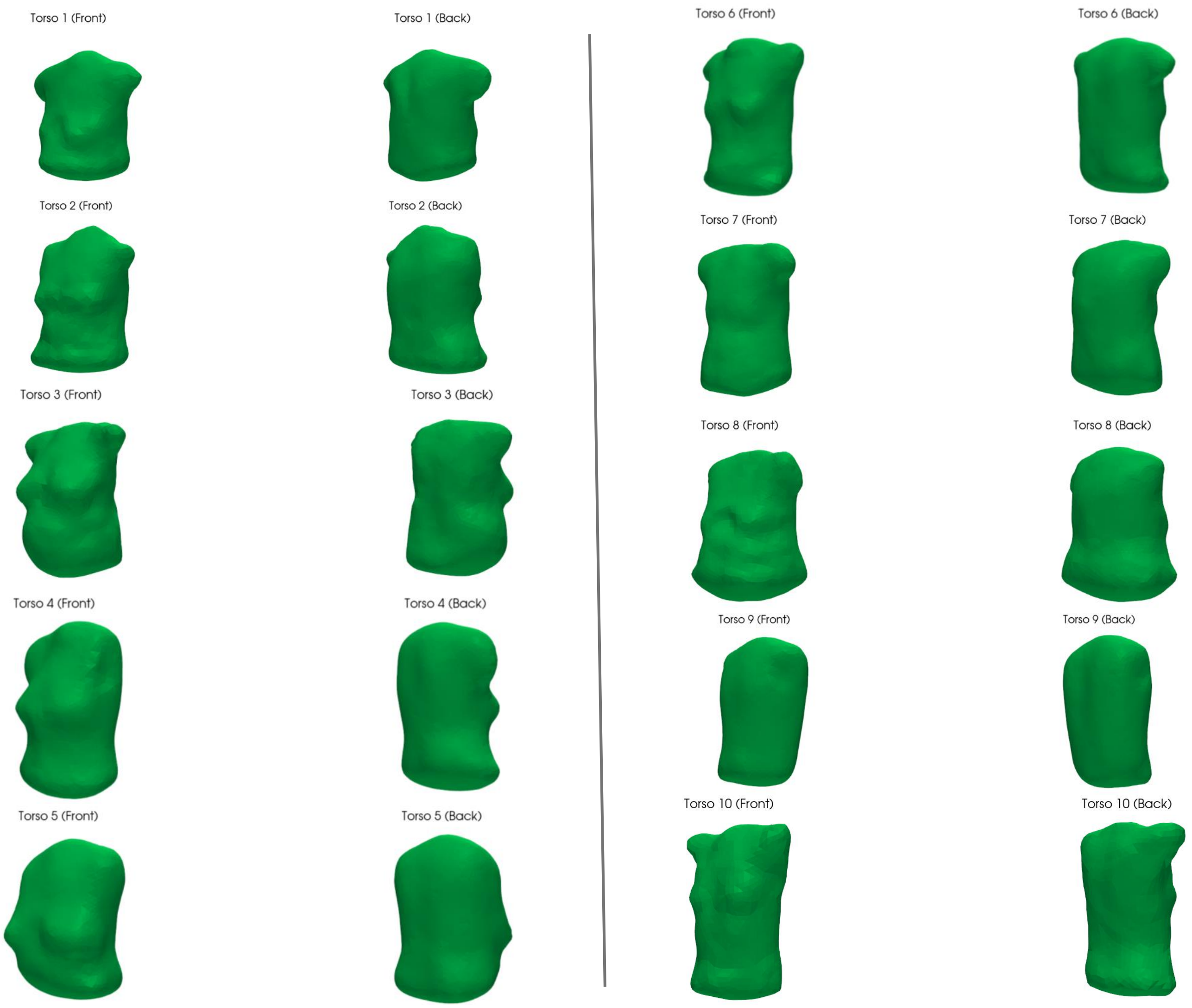

Figure S4. Geometries of 10 torsos used for the study. For each torso, the front (anterior) and back (posterior) of the torso is shown.

REFERENCES

Pedrón-Torrecilla, J., Rodrigo, M., Climent, A. M., Liberos, A., Pérez-David, E., Bermejo, J., et al. (2016). Noninvasive estimation of epicardial dominant high-frequency regions during atrial fibrillation. *Journal of Cardiovascular Electrophysiology* 27, 435–442
